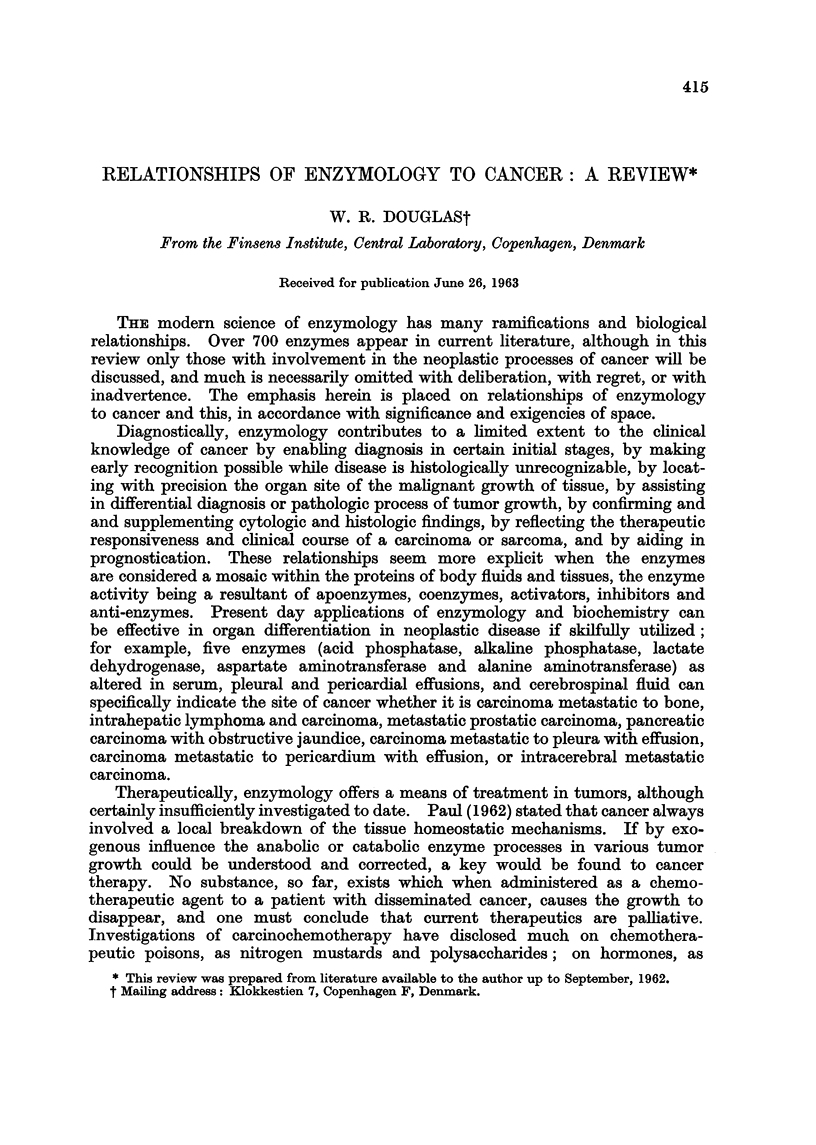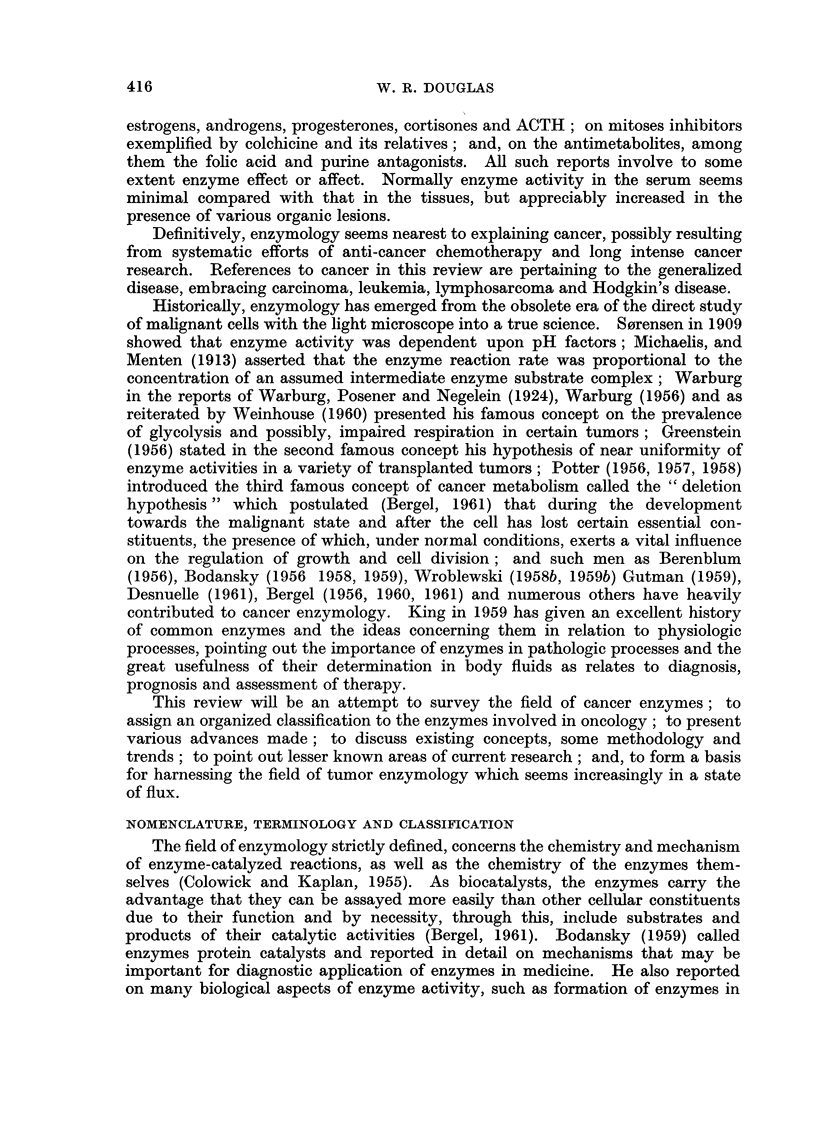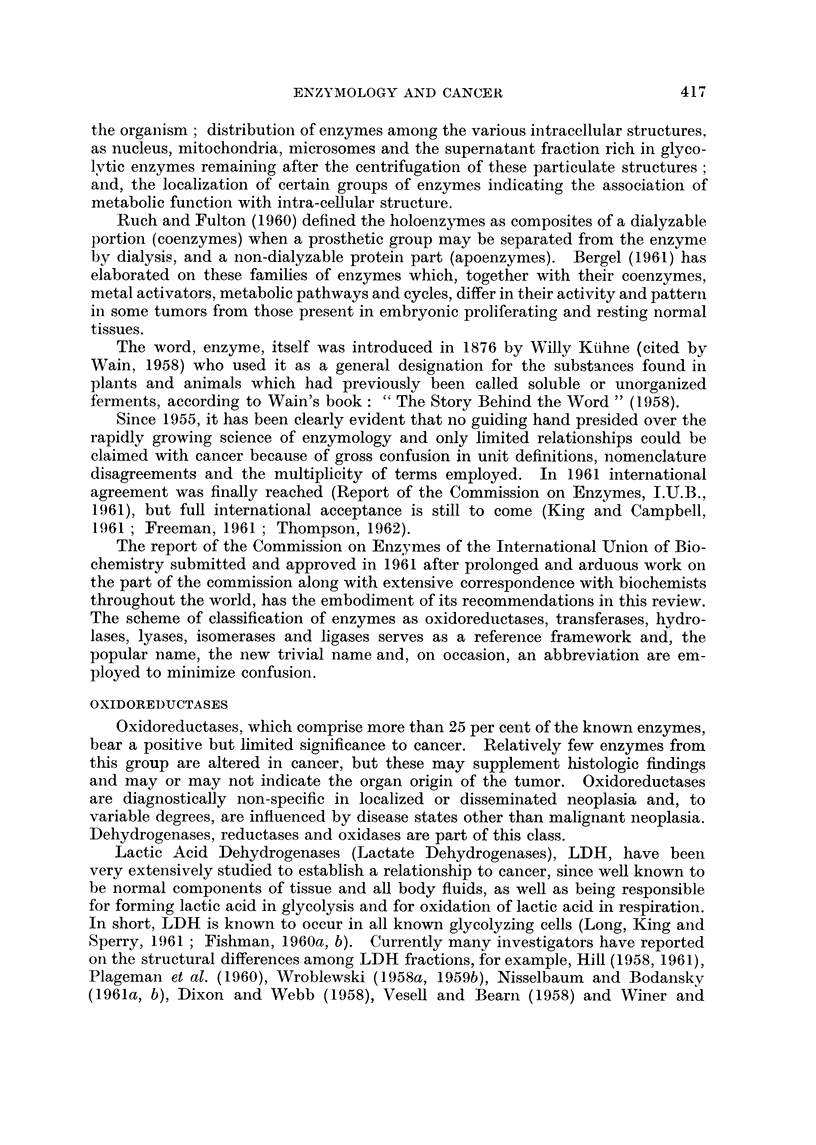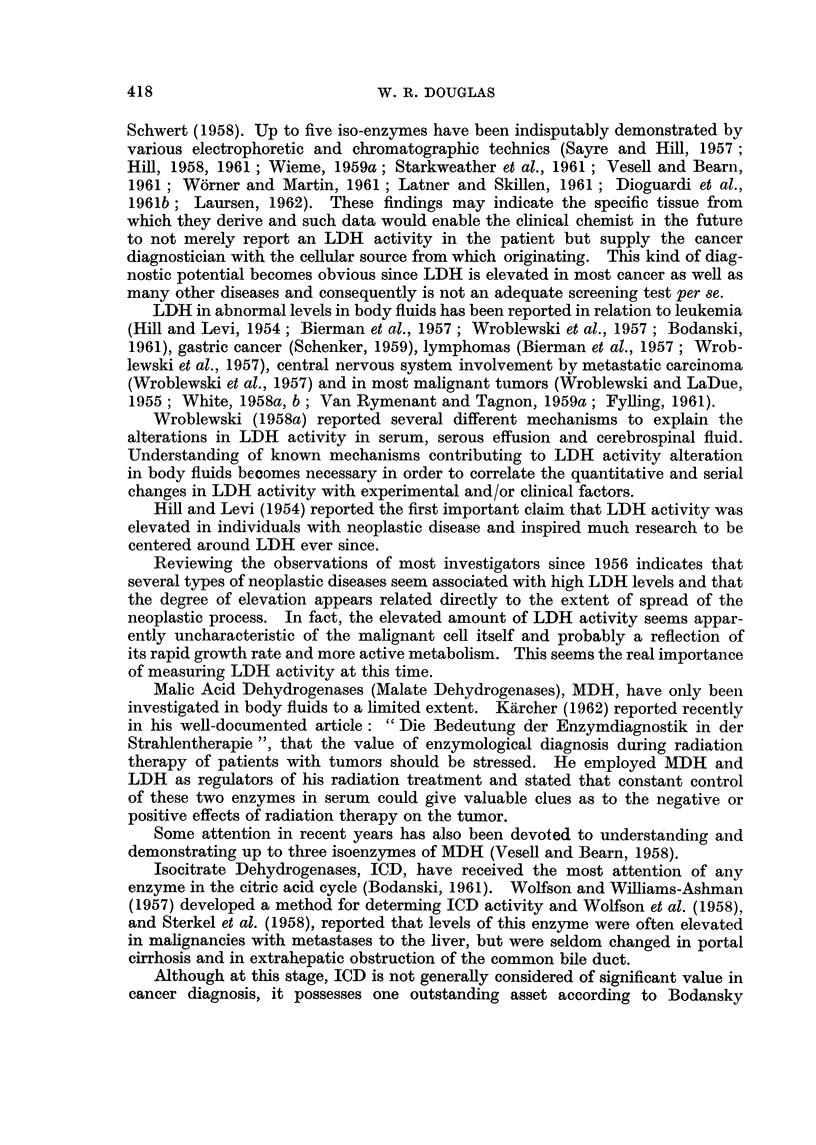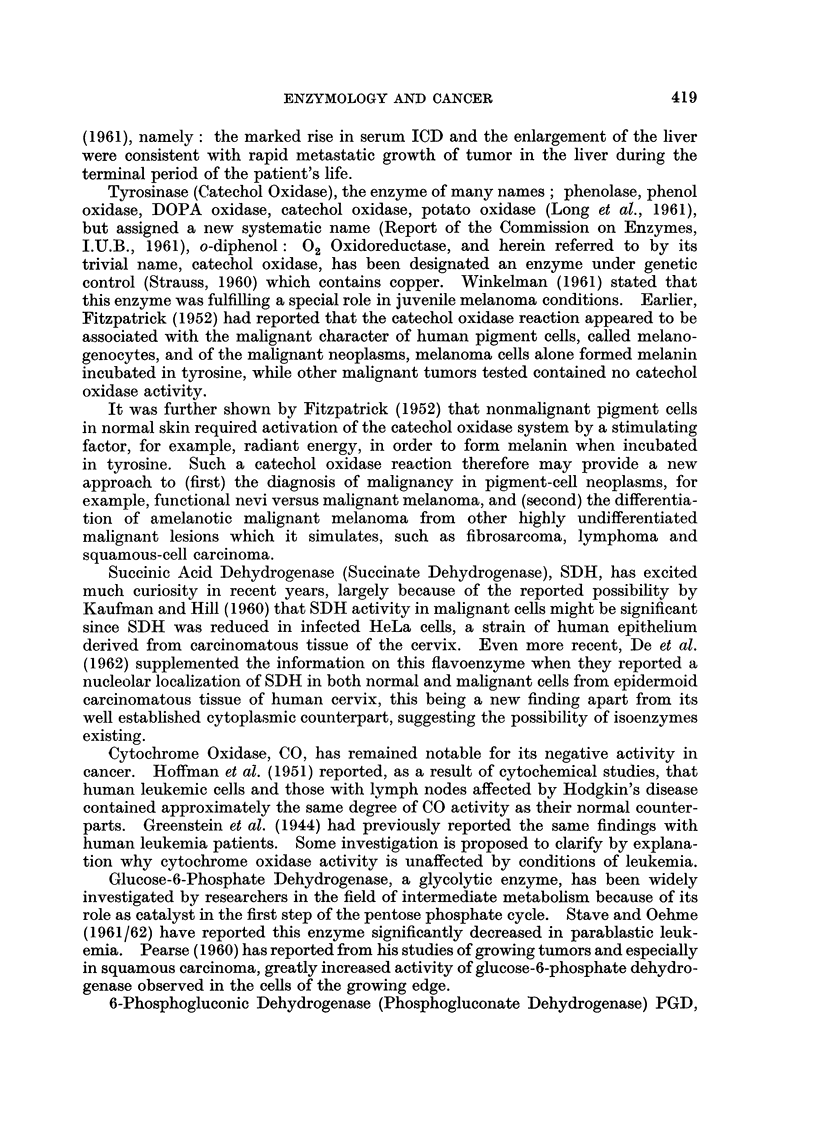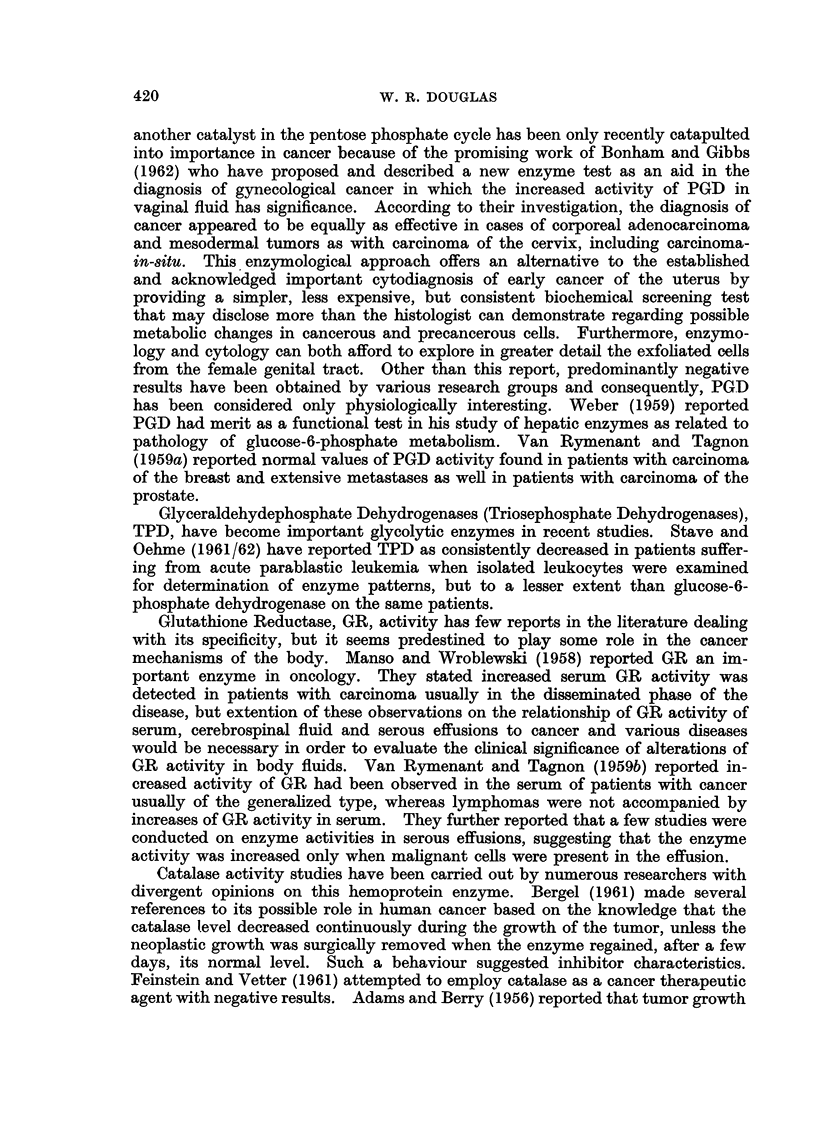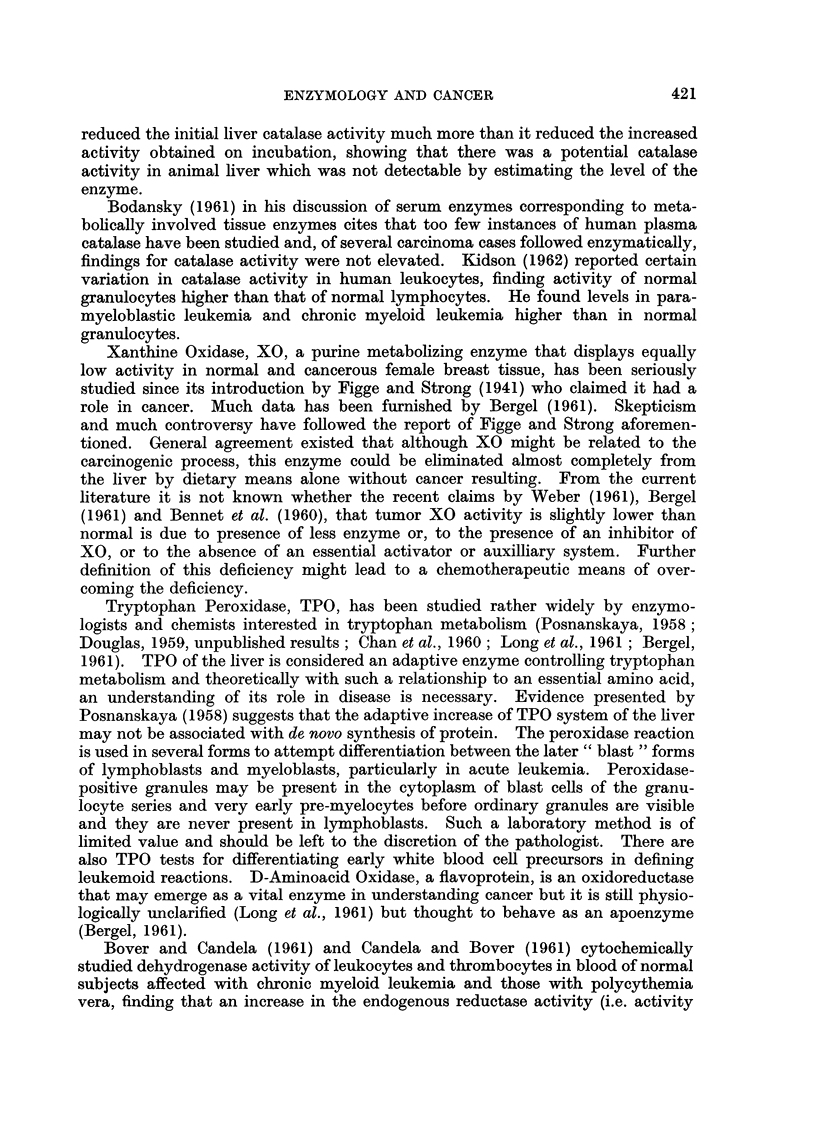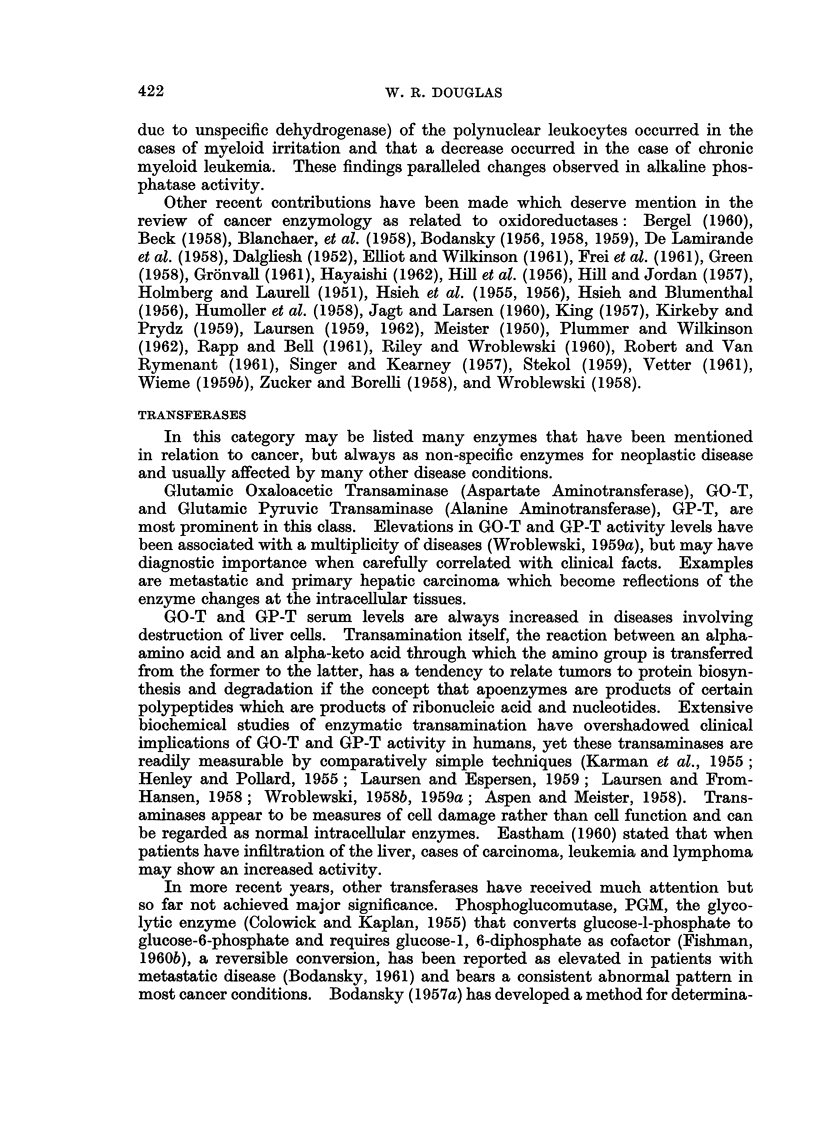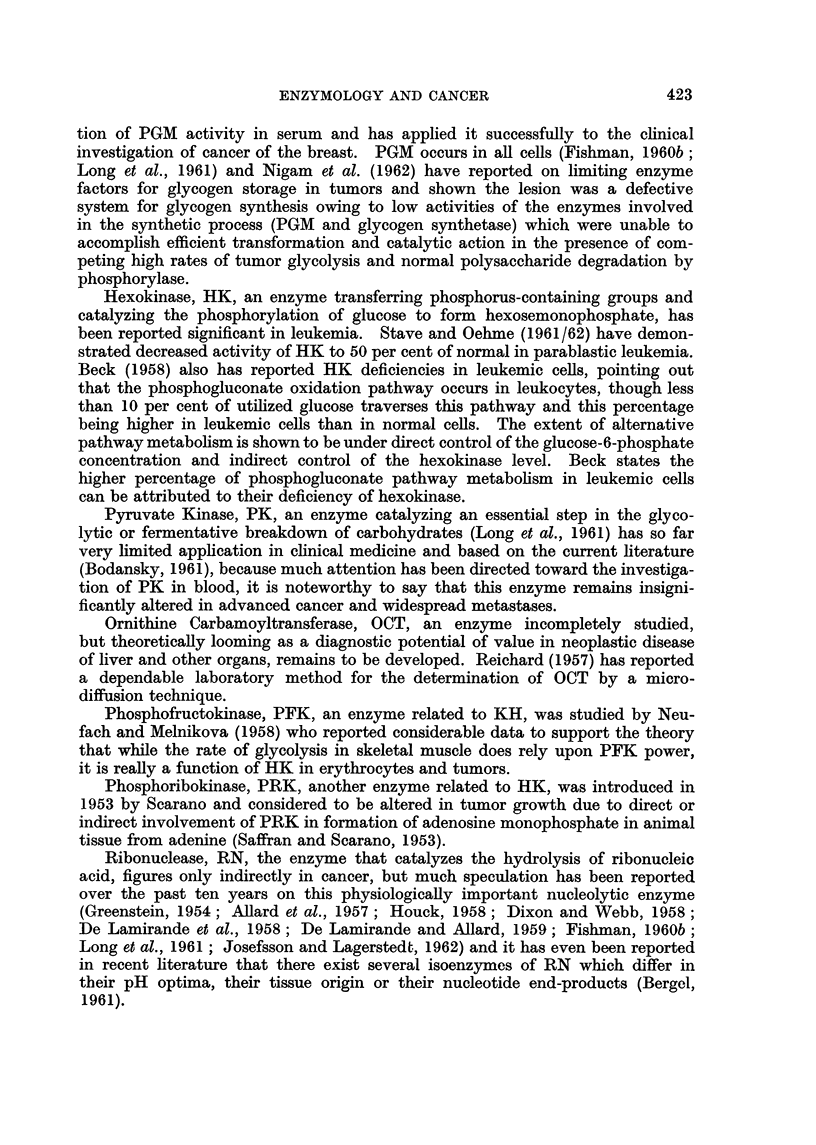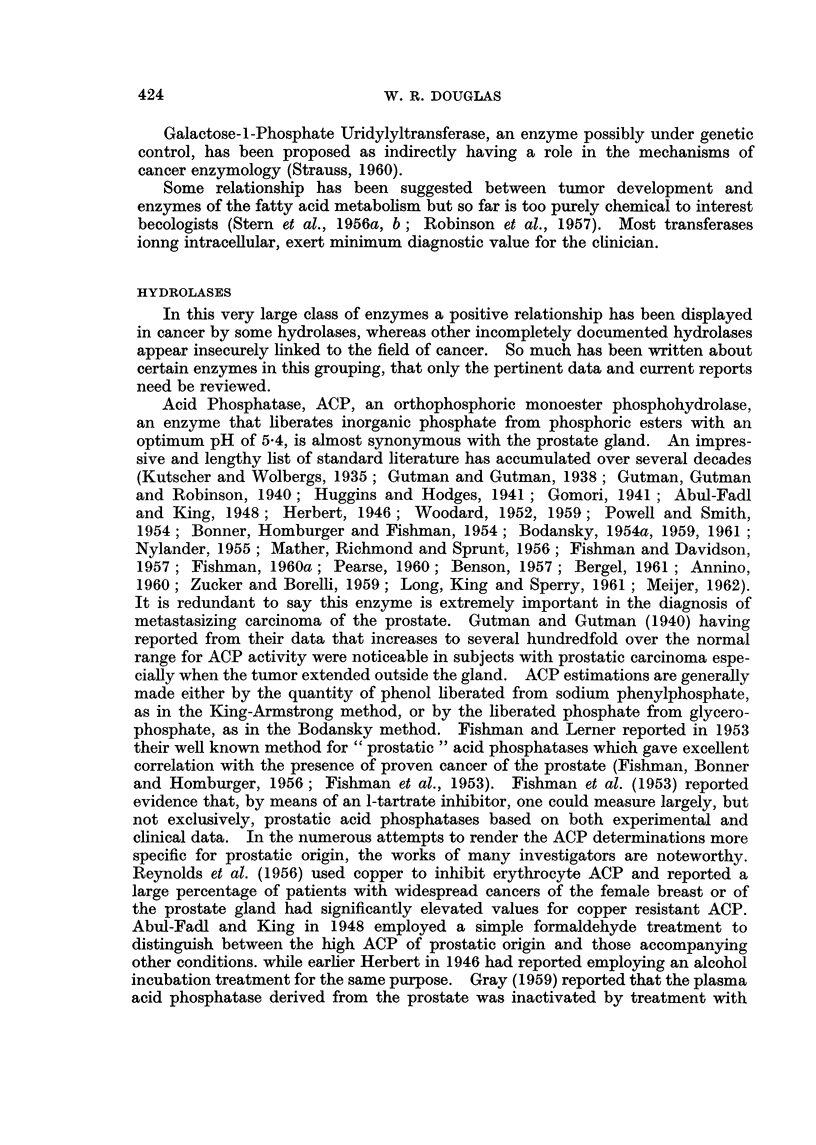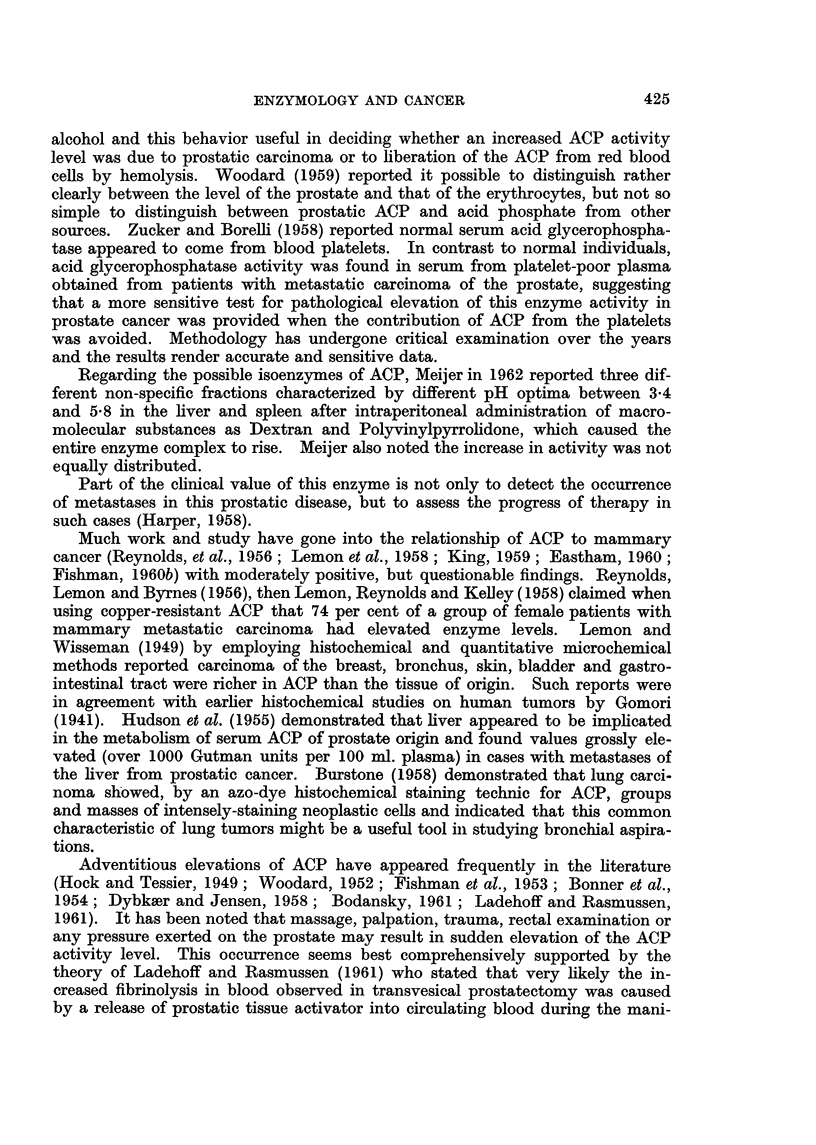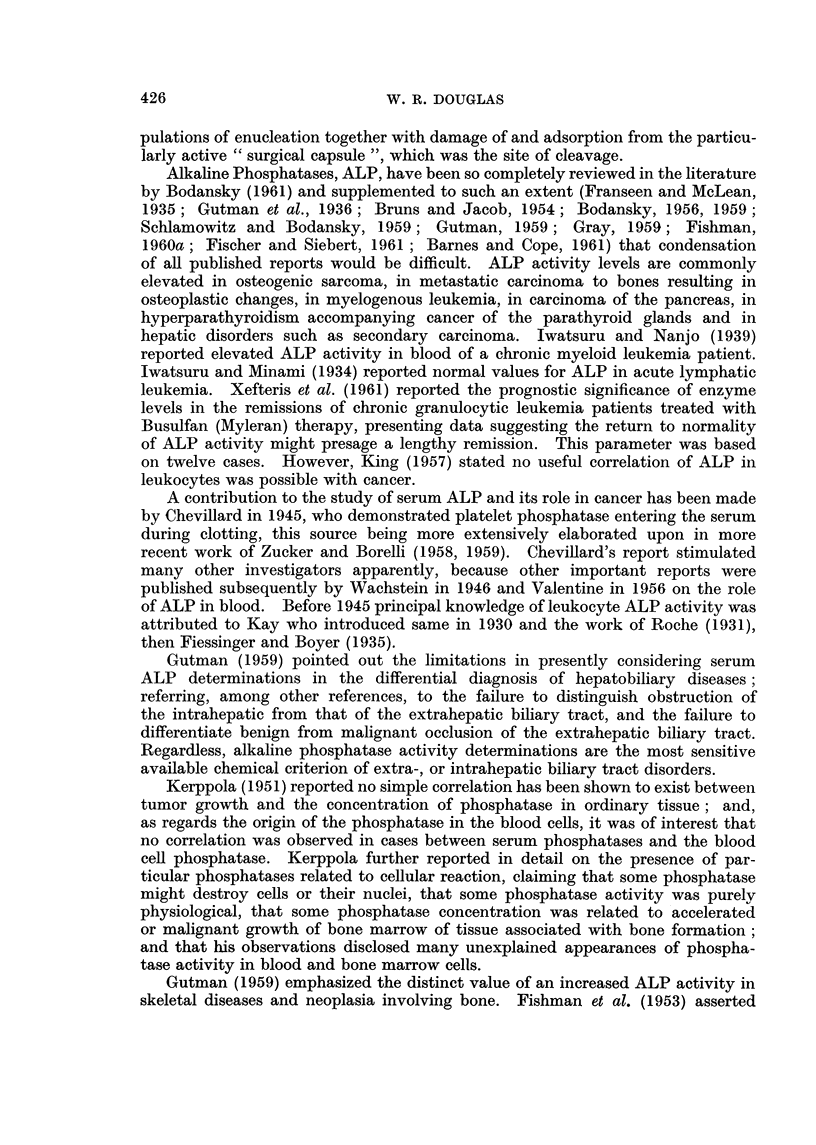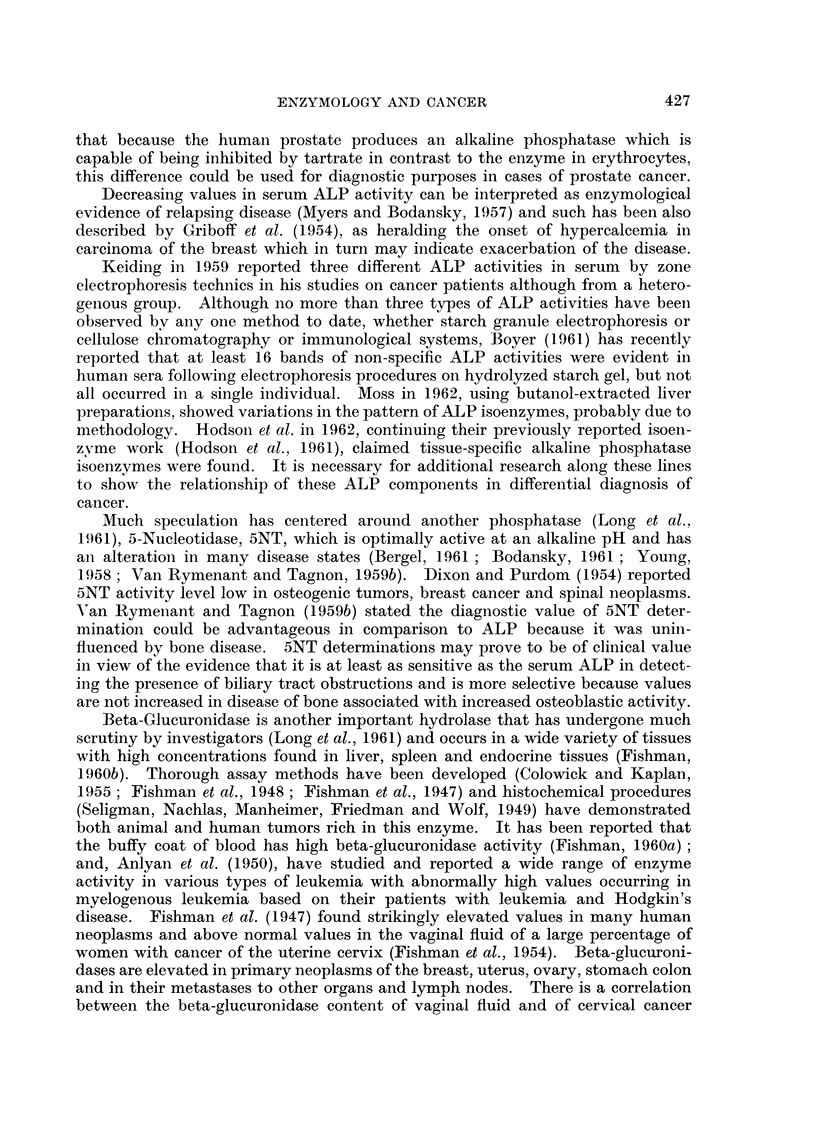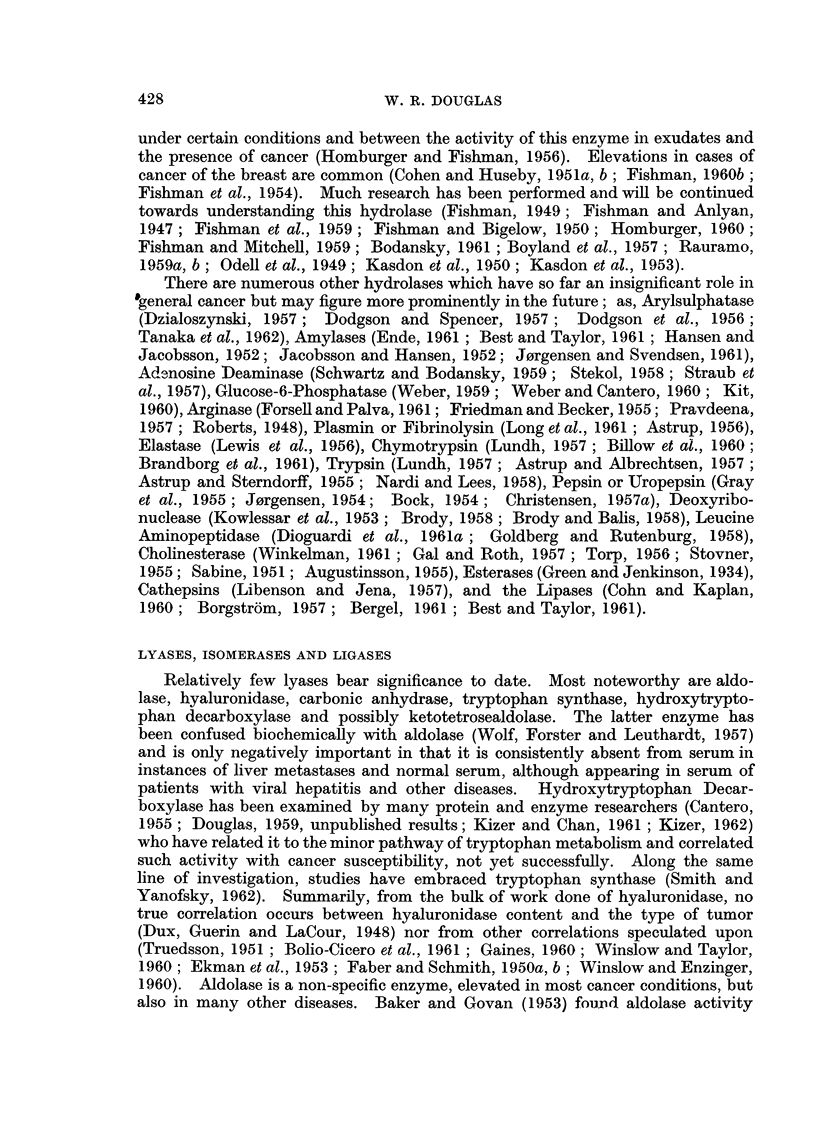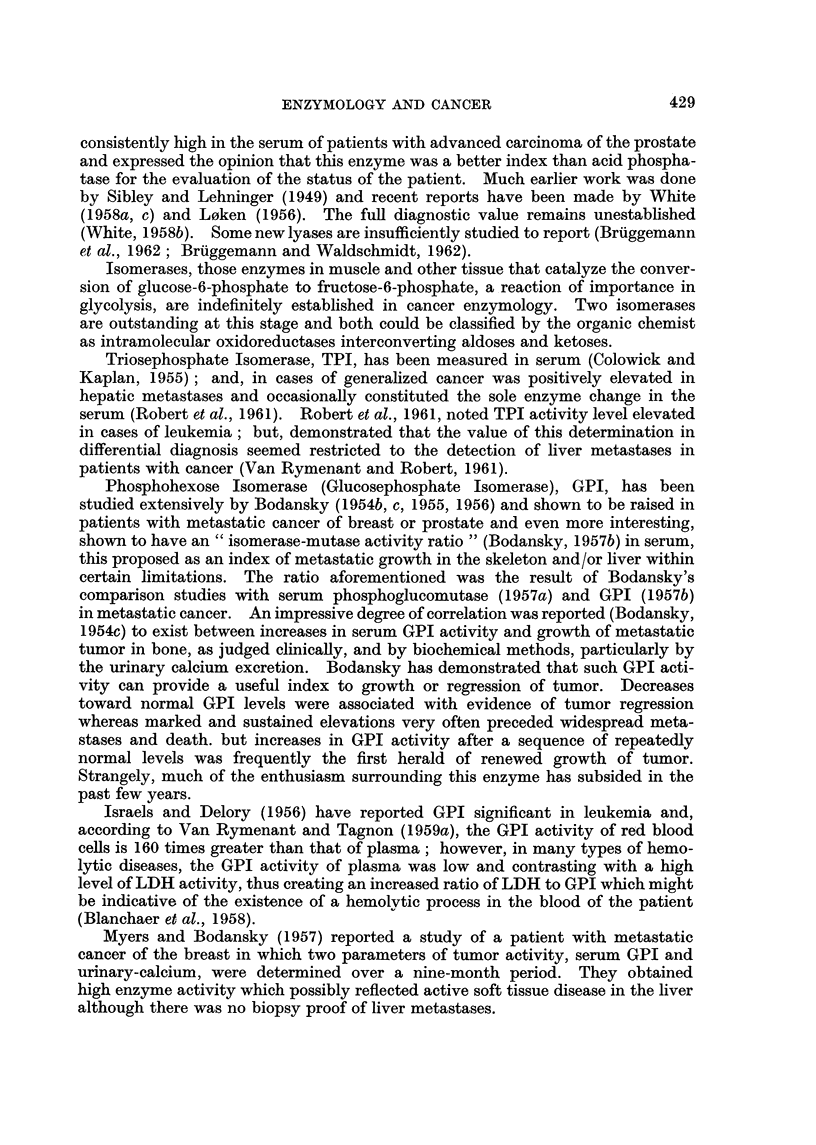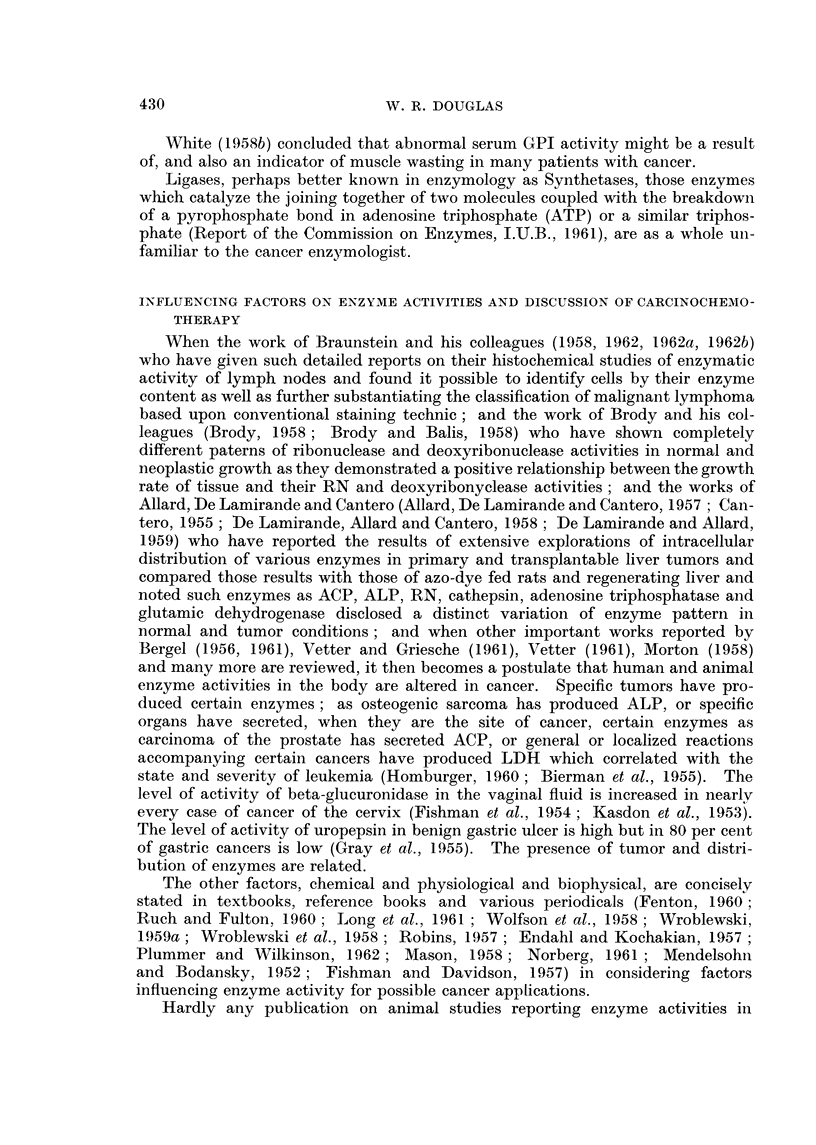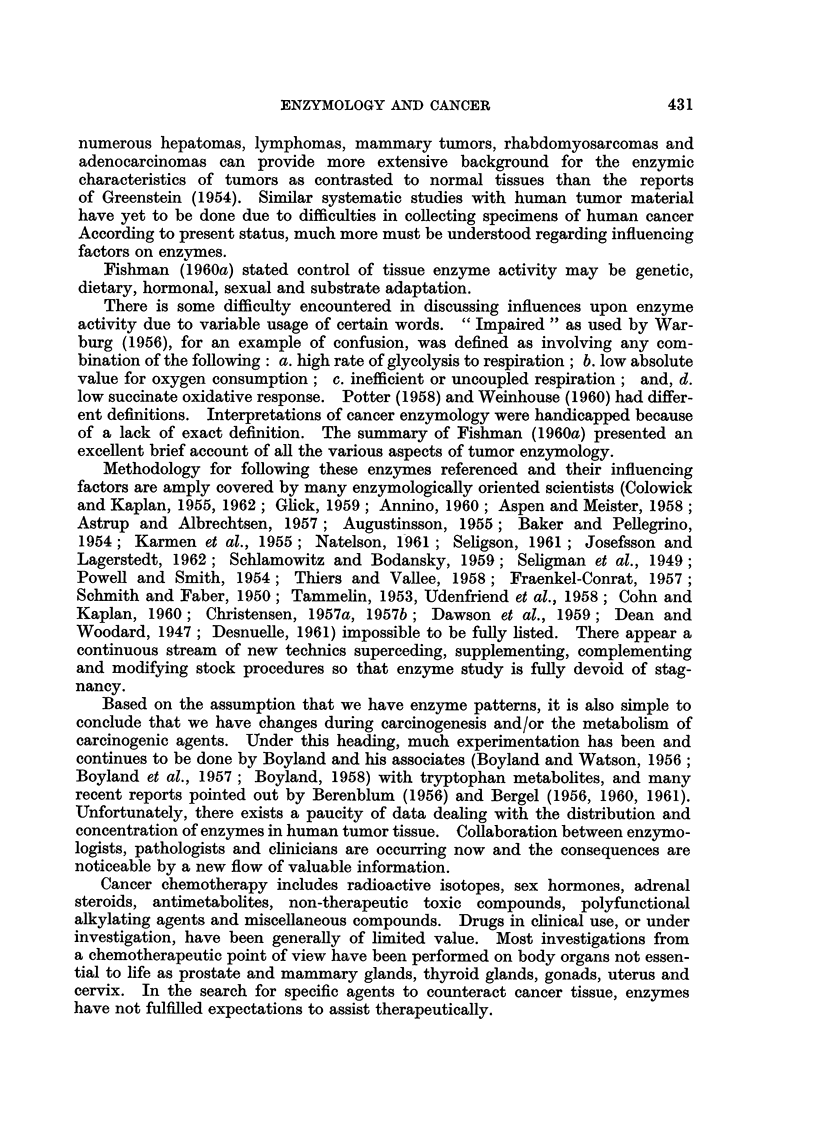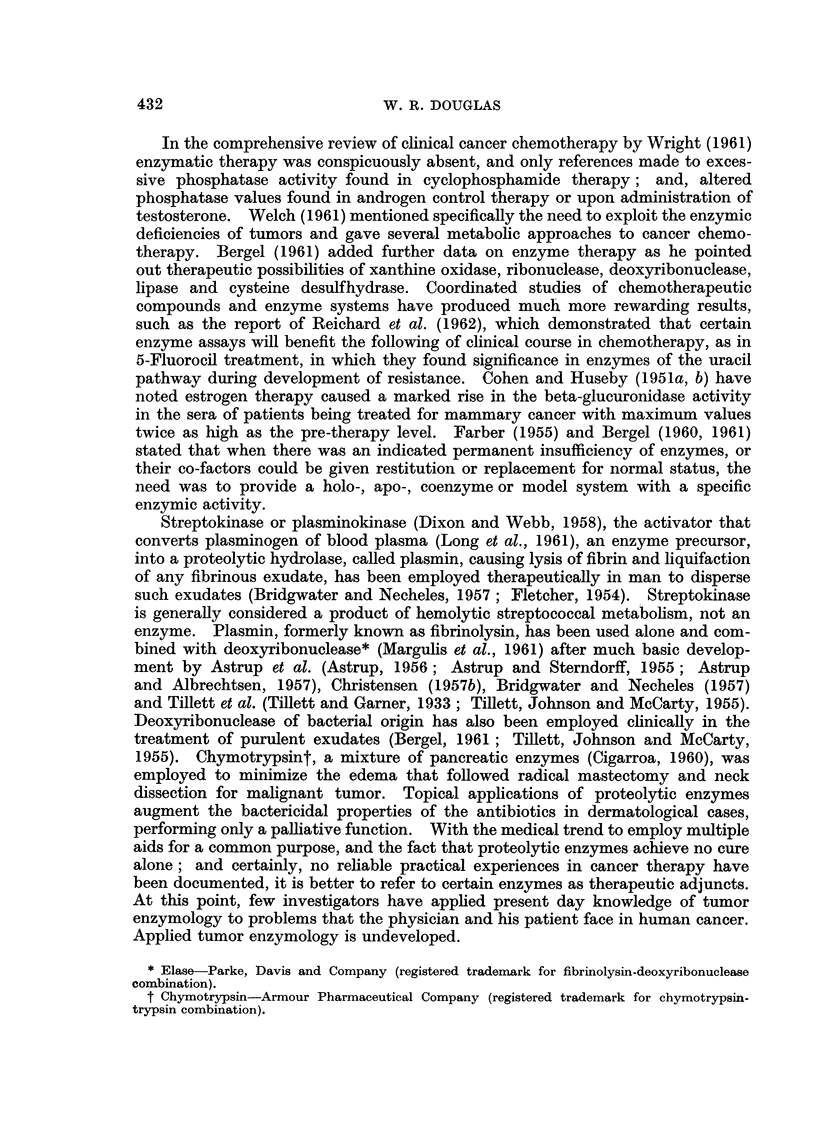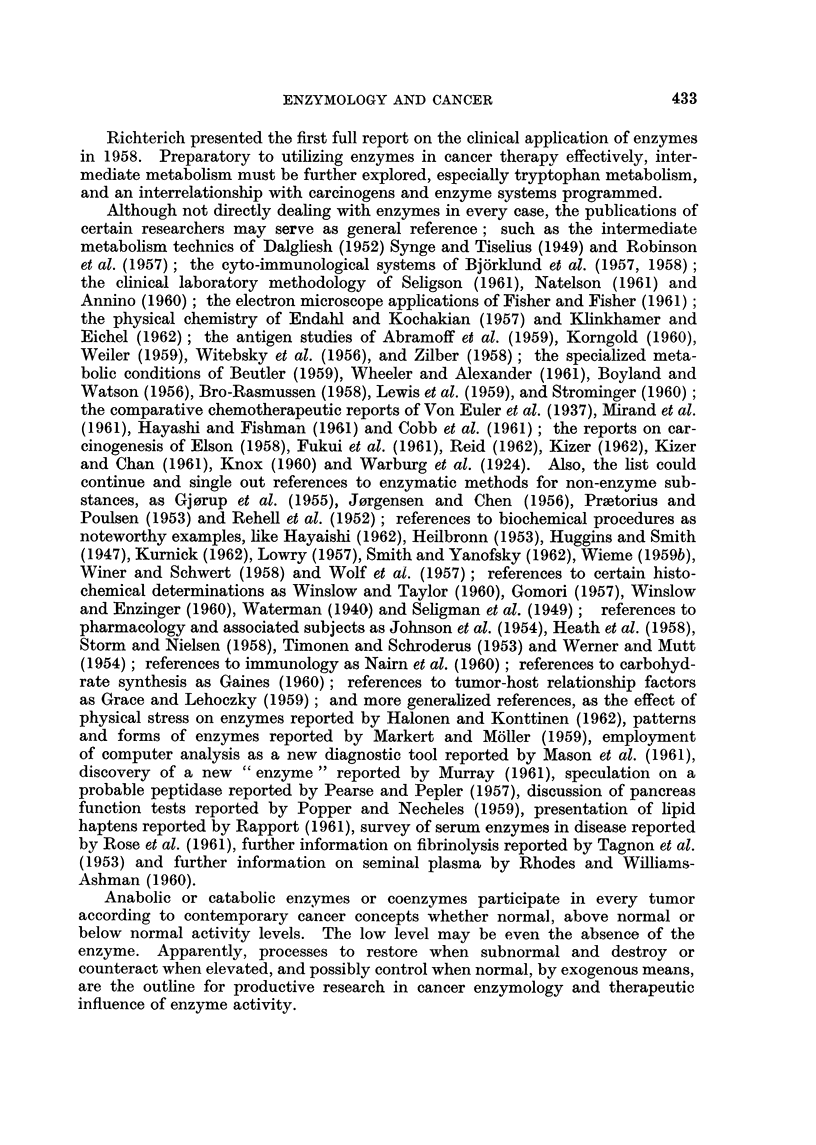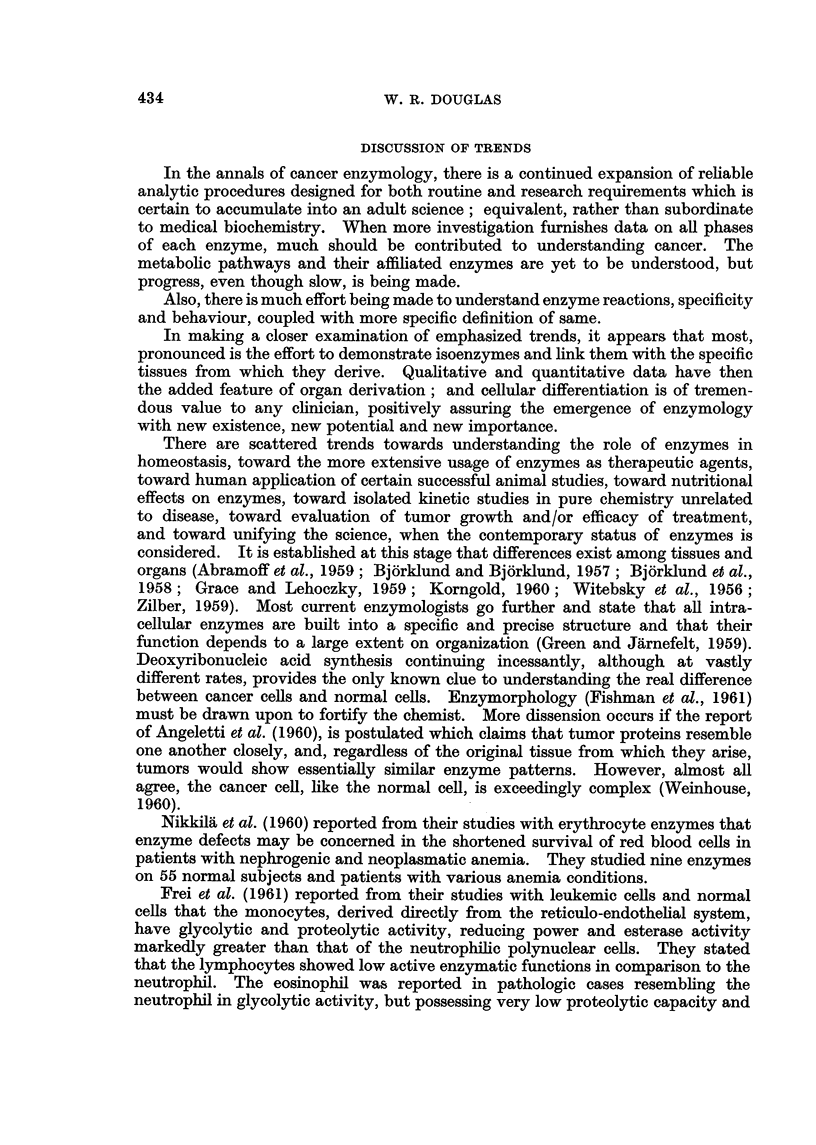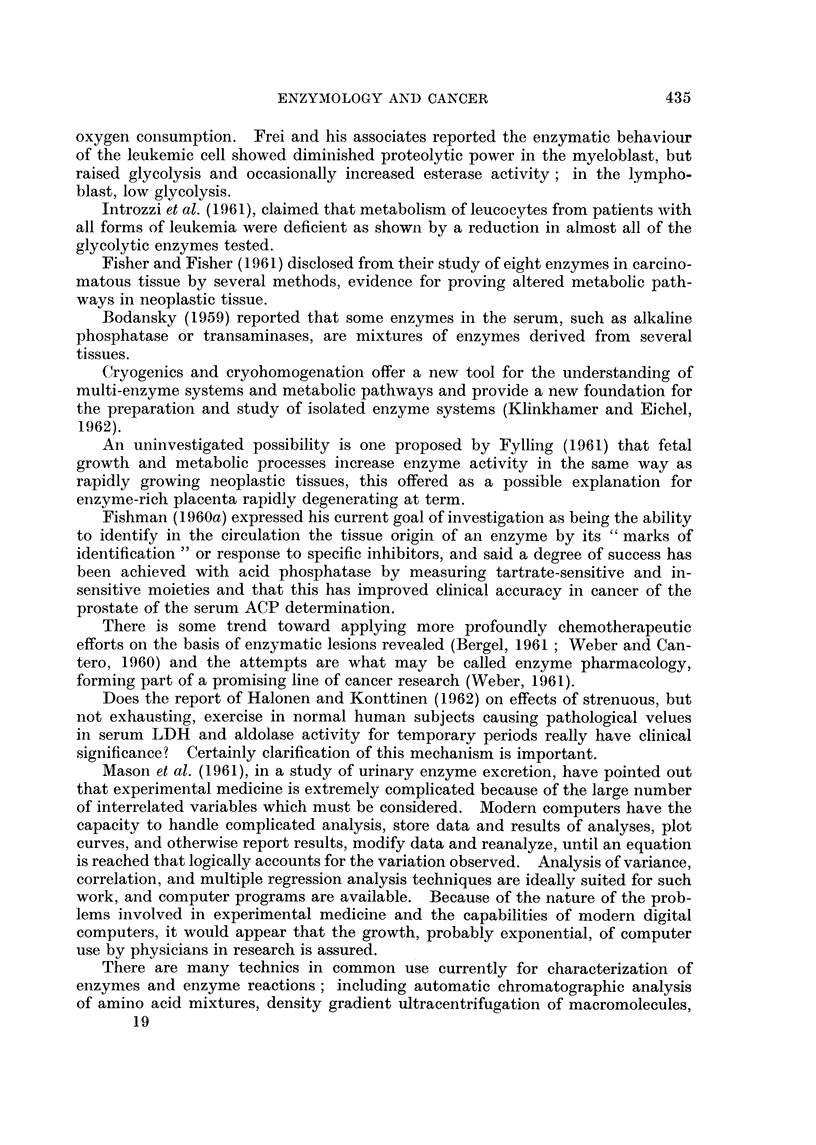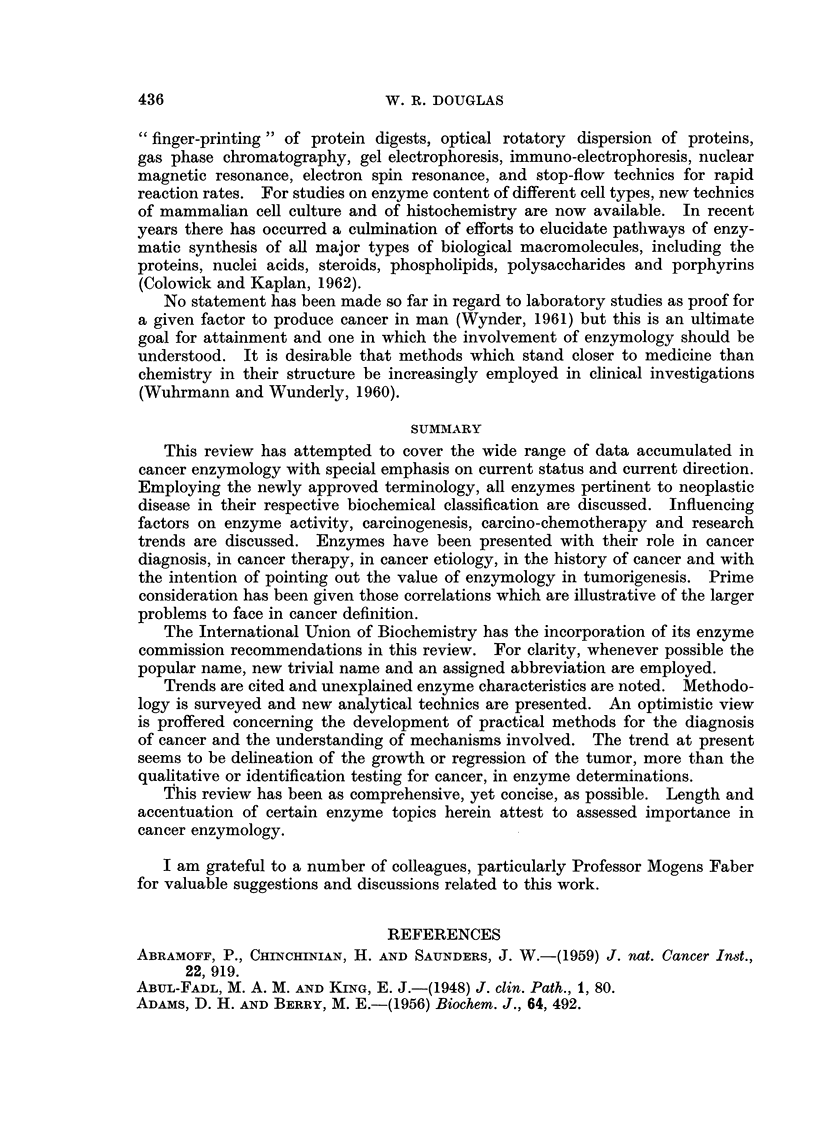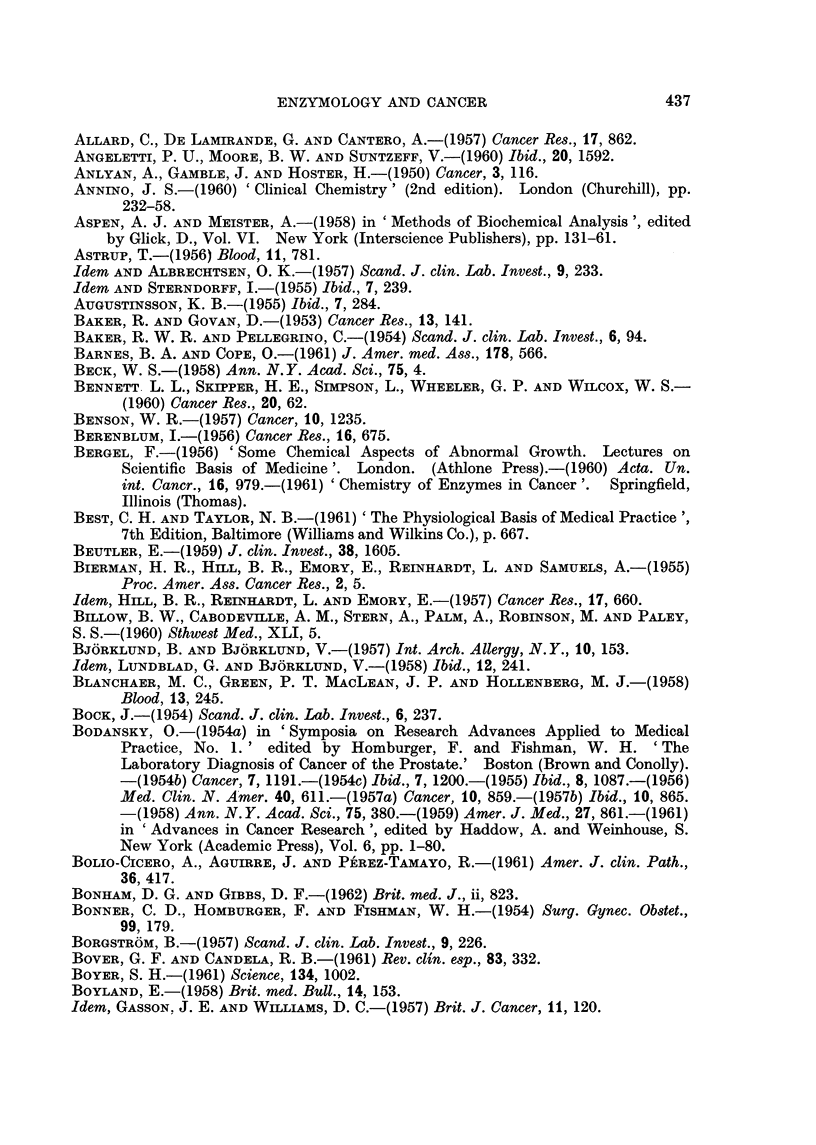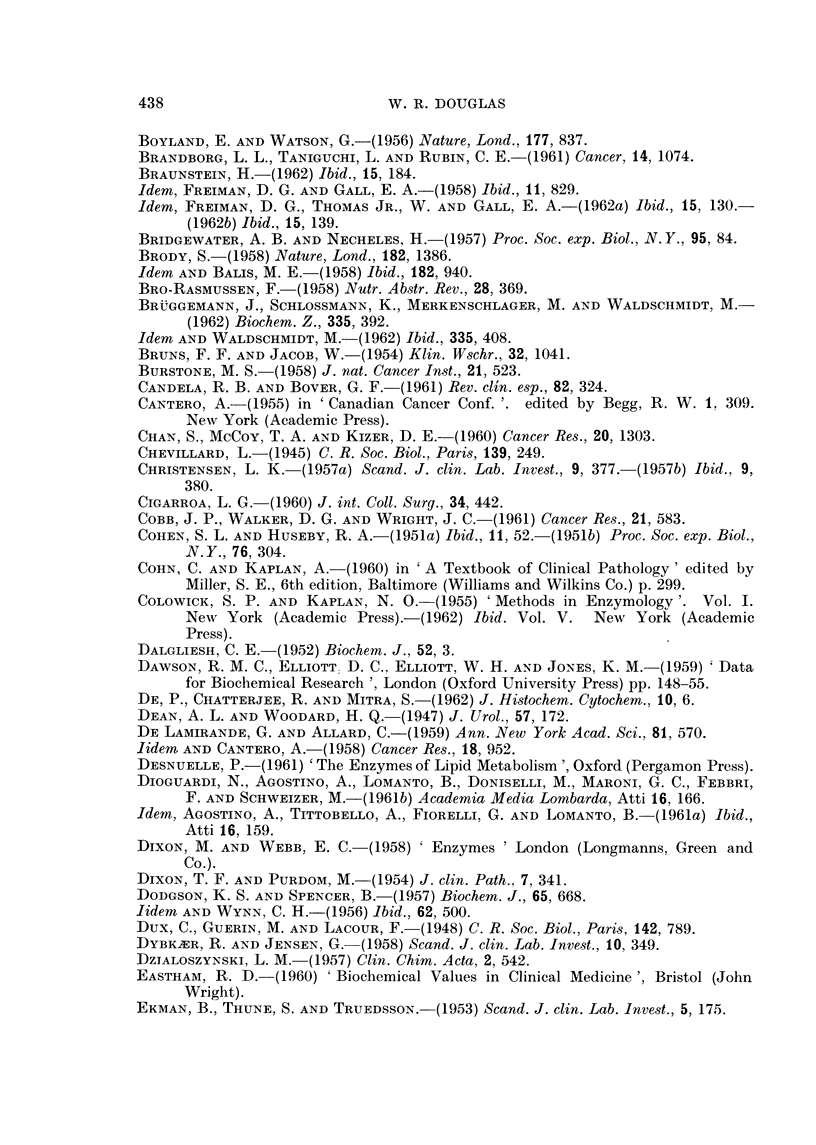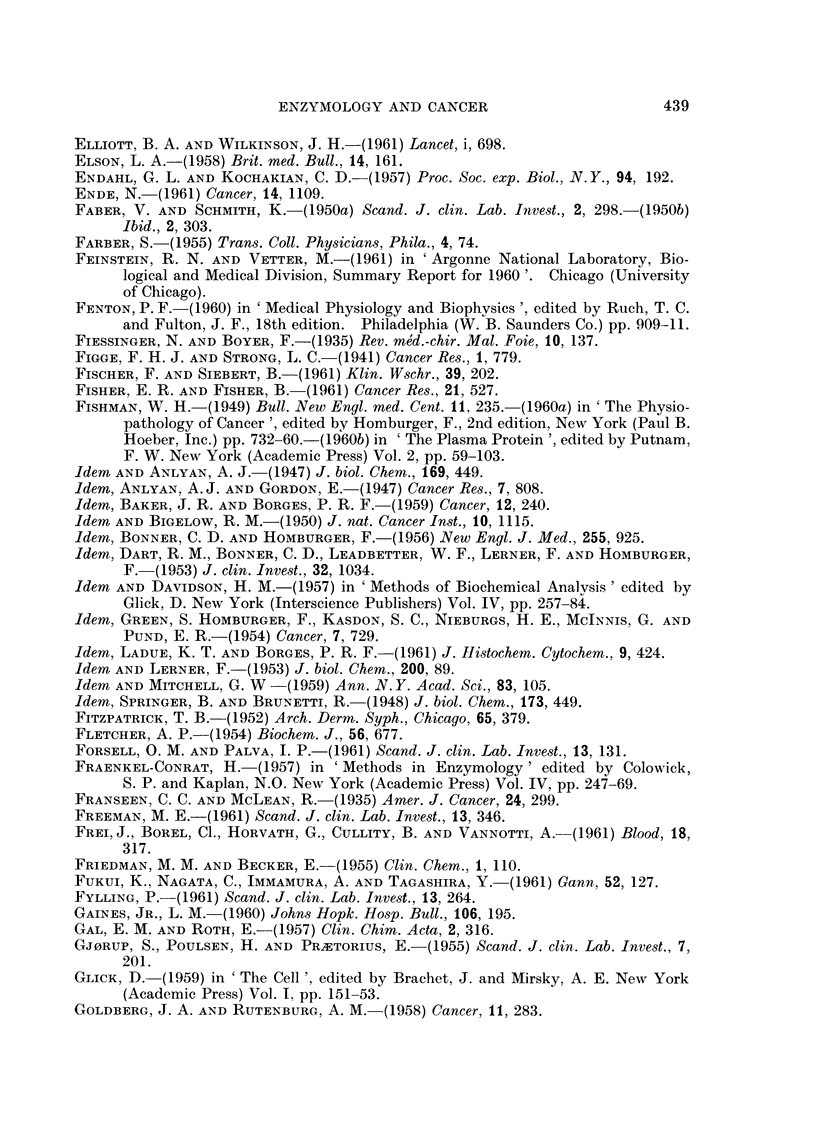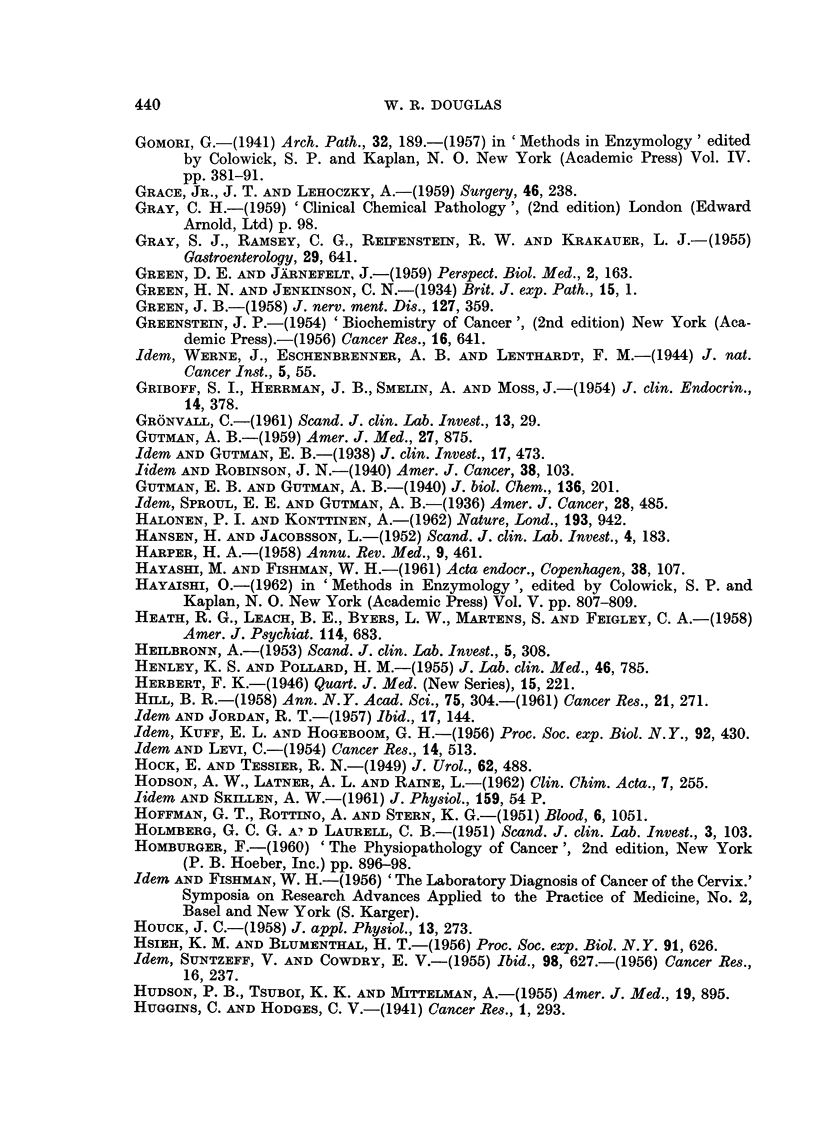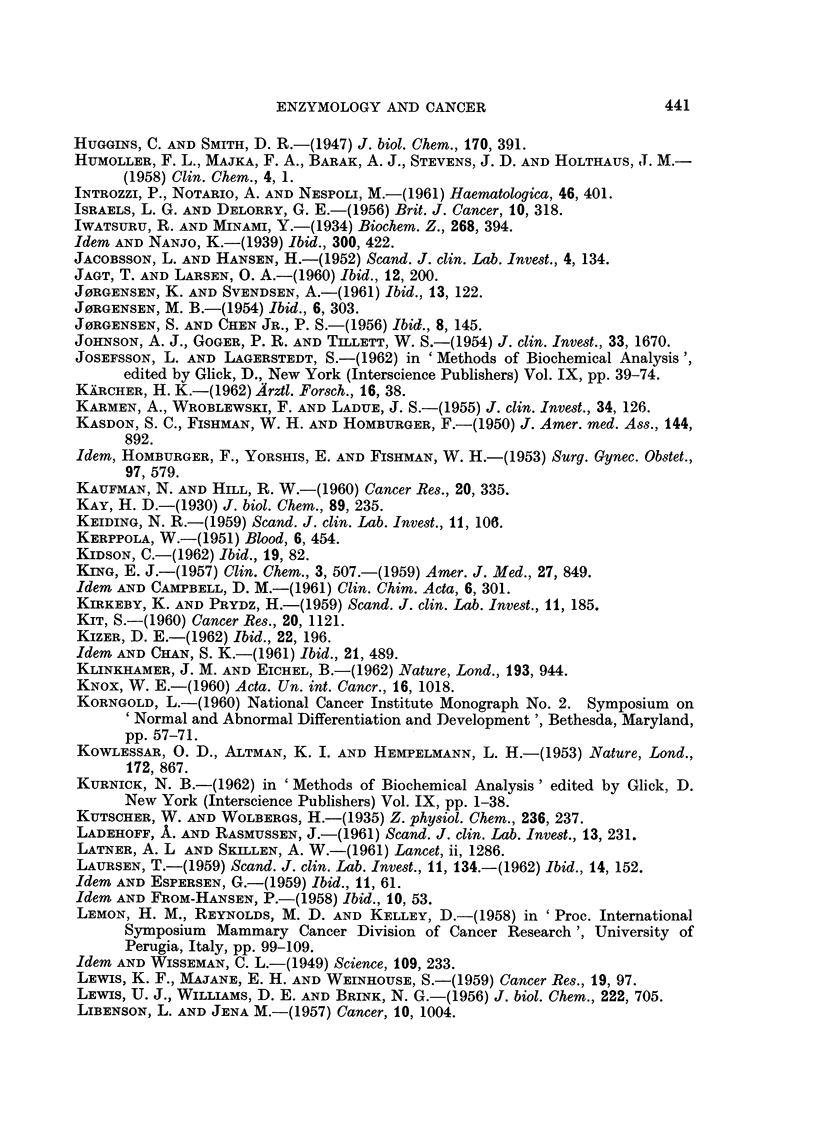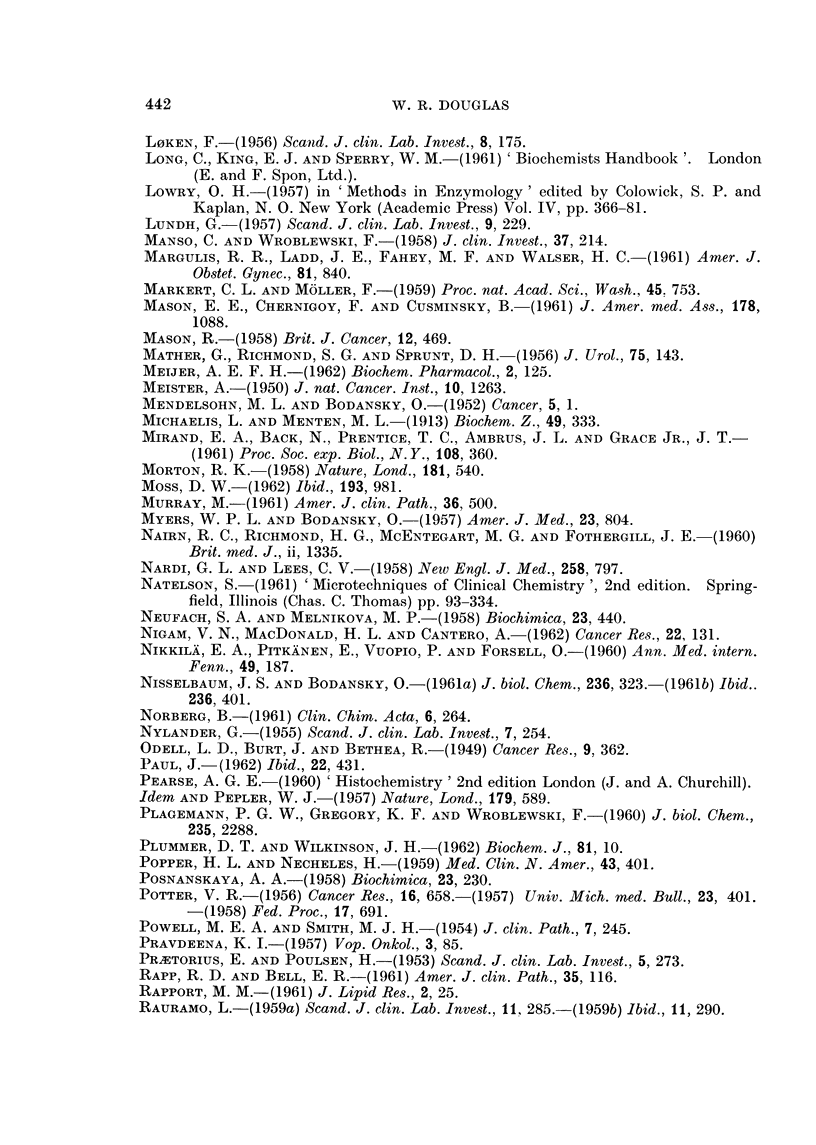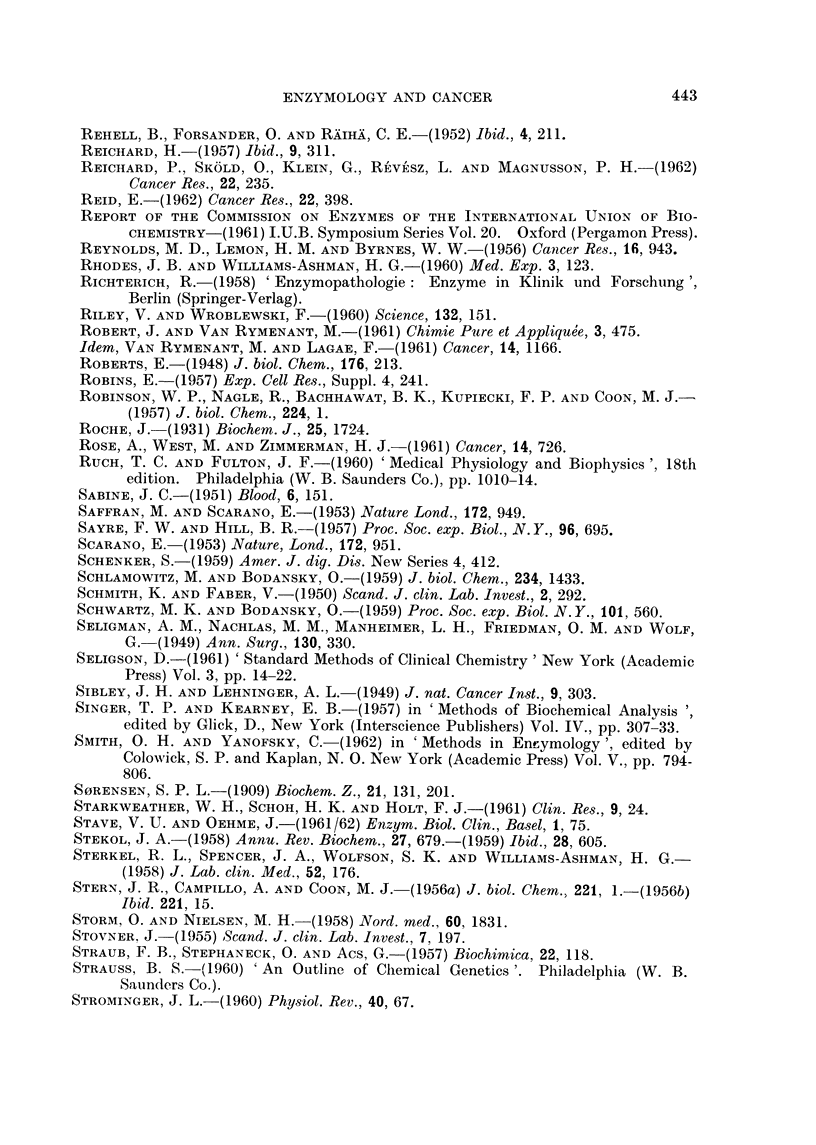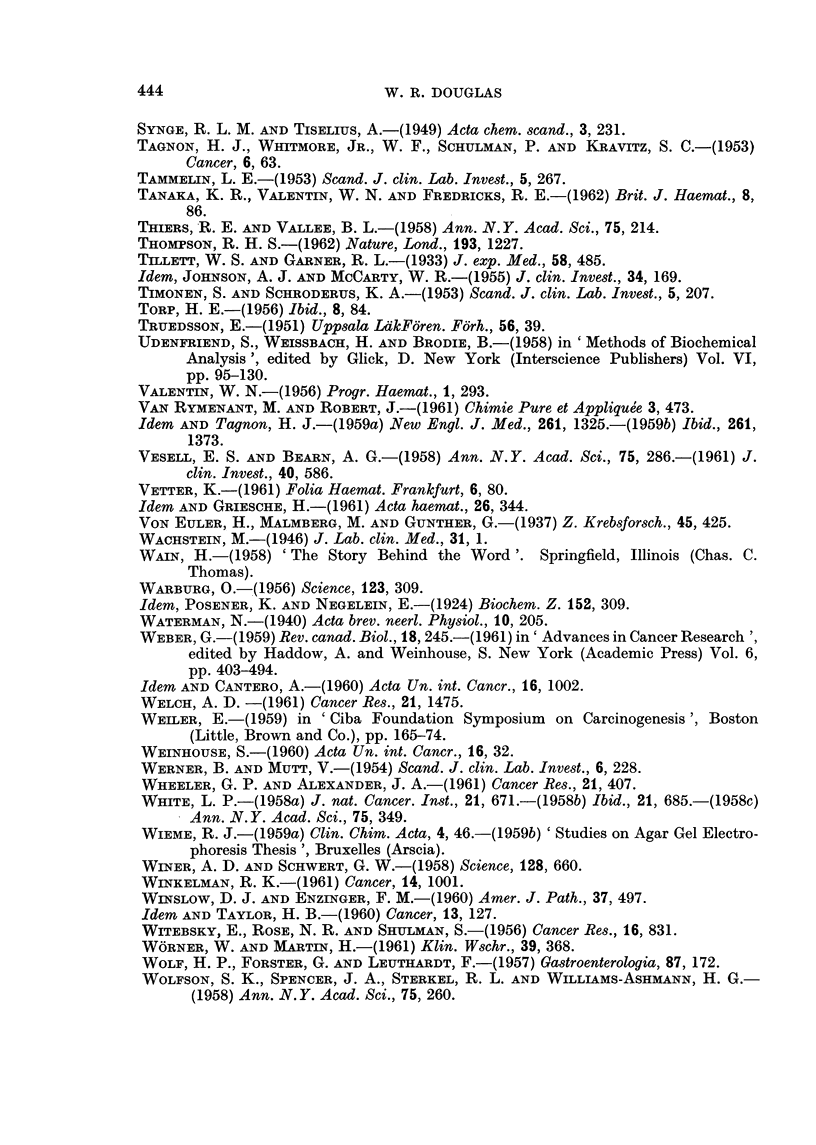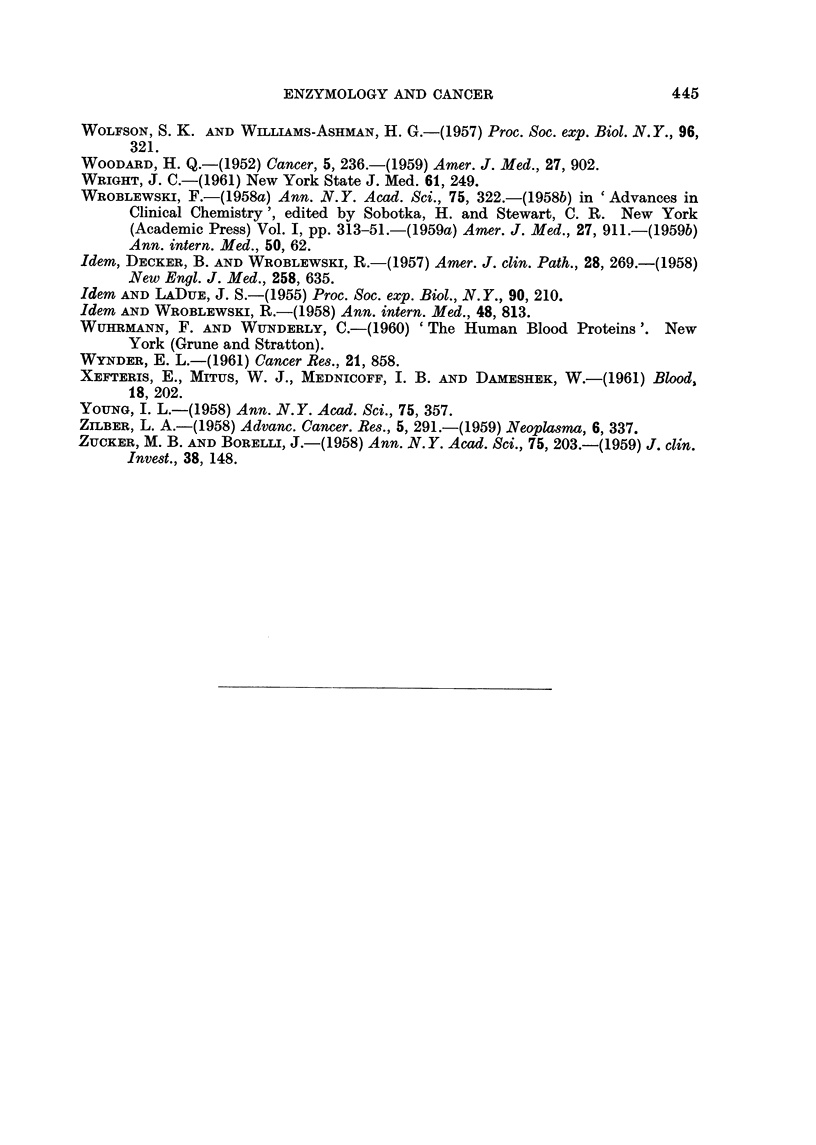# Relationships of Enzymology to Cancer: A Review*

**DOI:** 10.1038/bjc.1963.58

**Published:** 1963-09

**Authors:** W. R. Douglas


### RELATIONSHIPS OF ENZYMOLOGY TO CANCER : A REVIEW*

W. R. DOUGLAS†

*From the Finsens Institute, Central Laboratory, Copenhagen, Denmark*

Received for publication June 26, 1963

THE modern science of enzymology has many ramifications and biological relationships. Over 700 enzymes appear in current literature, although in this review only those with involvement in the neoplastic processes of cancer will be discussed, and much is necessarily omitted with deliberation, with regret, or with inadvertence. The emphasis herein is placed on relationships of enzymology to cancer and this, in accordance with significance and exigencies of space.

Diagnostically, enzymology contributes to a limited extent to the clinical knowledge of cancer by enabling diagnosis in certain initial stages, by making early recognition possible while disease is histologically unrecognizable, by locating with precision the organ site of the malignant growth of tissue, by assisting in differential diagnosis or pathologic process of tumor growth, by confirming and and supplementing cytologic and histologic findings, by reflecting the therapeutic responsiveness and clinical course of a carcinoma or sarcoma, and by aiding in prognostication. These relationships seem more explicit when the enzymes are considered a mosaic within the proteins of body fluids and tissues, the enzyme activity being a resultant of apoenzymes, coenzymes, activators, inhibitors and anti-enzymes. Present day applications of enzymology and biochemistry can be effective in organ differentiation in neoplastic disease if skilfully utilized ; for example, five enzymes (acid phosphatase, alkaline phosphatase, lactate dehydrogenase, aspartate aminotransferase and alanine aminotransferase) as altered in serum, pleural and pericardial effusions, and cerebrospinal fluid can specifically indicate the site of cancer whether it is carcinoma metastatic to bone, intrahepatic lymphoma and carcinoma, metastatic prostatic carcinoma, pancreatic carcinoma with obstructive jaundice, carcinoma metastatic to pleura with effusion, carcinoma metastatic to pericardium with effusion, or intracerebral metastatic carcinoma.

Therapeutically, enzymology offers a means of treatment in tumors, although certainly insufficiently investigated to date. Paul (1962) stated that cancer always involved a local breakdown of the tissue homeostatic mechanisms. If by exogenous influence the anabolic or catabolic enzyme processes in various tumor growth could be understood and corrected, a key would be found to cancer therapy. No substance, so far, exists which when administered as a chemotherapeutic agent to a patient with disseminated cancer, causes the growth to disappear, and one must conclude that current therapeutics are palliative. Investigations of carcinochemotherapy have disclosed much on chemotherapeutic poisons, as nitrogen mustards and polysaccharides ; on hormones, as

* This review was prepared from literature available to the author up to September, 1962.
† Mailing address : Klokkestien 7, Copenhagen F, Denmark.

estrogens, androgens, progesterones, cortisones and ACTH ; on mitoses inhibitors exemplified by colchicine and its relatives ; and, on the antimetabolites, among them the folic acid and purine antagonists. All such reports involve to some extent enzyme effect or affect. Normally enzyme activity in the serum seems minimal compared with that in the tissues, but appreciably increased in the presence of various organic lesions.

Definitively, enzymology seems nearest to explaining cancer, possibly resulting from systematic efforts of anti-cancer chemotherapy and long intense cancer research. References to cancer in this review are pertaining to the generalized disease, embracing carcinoma, leukemia, lymphosarcoma and Hodgkin's disease.

Historically, enzymology has emerged from the obsolete era of the direct study of malignant cells with the light microscope into a true science. Sørensen in 1909 showed that enzyme activity was dependent upon pH factors ; Michaelis, and Menten (1913) asserted that the enzyme reaction rate was proportional to the concentration of an assumed intermediate enzyme substrate complex ; Warburg in the reports of Warburg, Posener and Negelein (1924), Warburg (1956) and as reiterated by Weinhouse (1960) presented his famous concept on the prevalence of glycolysis and possibly, impaired respiration in certain tumors ; Greenstein (1956) stated in the second famous concept his hypothesis of near uniformity of enzyme activities in a variety of transplanted tumors ; Potter (1956, 1957, 1958) introduced the third famous concept of cancer metabolism called the " deletion hypothesis " which postulated (Bergel, 1961) that during the development towards the malignant state and after the cell has lost certain essential constituents, the presence of which, under normal conditions, exerts a vital influence on the regulation of growth and cell division ; and such men as Berenblum (1956), Bodansky (1956 1958, 1959), Wroblewski (1958*b*, 1959*b*) Gutman (1959), Desnuelle (1961), Bergel (1956, 1960, 1961) and numerous others have heavily contributed to cancer enzymology. King in 1959 has given an excellent history of common enzymes and the ideas concerning them in relation to physiologic processes, pointing out the importance of enzymes in pathologic processes and the great usefulness of their determination in body fluids as relates to diagnosis, prognosis and assessment of therapy.

This review will be an attempt to survey the field of cancer enzymes ; to assign an organized classification to the enzymes involved in oncology ; to present various advances made ; to discuss existing concepts, some methodology and trends ; to point out lesser known areas of current research ; and, to form a basis for harnessing the field of tumor enzymology which seems increasingly in a state of flux.

### NOMENCLATURE, TERMINOLOGY AND CLASSIFICATION

The field of enzymology strictly defined, concerns the chemistry and mechanism of enzyme-catalyzed reactions, as well as the chemistry of the enzymes themselves (Colowick and Kaplan, 1955). As biocatalysts, the enzymes carry the advantage that they can be assayed more easily than other cellular constituents due to their function and by necessity, through this, include substrates and products of their catalytic activities (Bergel, 1961). Bodansky (1959) called enzymes protein catalysts and reported in detail on mechanisms that may be important for diagnostic application of enzymes in medicine. He also reported on many biological aspects of enzyme activity, such as formation of enzymes in the organism ; distribution of enzymes among the various intracellular structures, as nucleus, mitochondria, microsomes and the supernatant fraction rich in glycolytic enzymes remaining after the centrifugation of these particulate structures ; and, the localization of certain groups of enzymes indicating the association of metabolic function with intra-cellular structure.

Ruch and Fulton (1960) defined the holoenzymes as composites of a dialyzable portion (coenzymes) when a prosthetic group may be separated from the enzyme by dialysis, and a non-dialyzable protein part (apoenzymes). Bergel (1961) has elaborated on these families of enzymes which, together with their coenzymes, metal activators, metabolic pathways and cycles, differ in their activity and pattern in some tumors from those present in embryonic proliferating and resting normal tissues.

The word, enzyme, itself was introduced in 1876 by Willy Kühne (cited by Wain, 1958) who used it as a general designation for the substances found in plants and animals which had previously been called soluble or unorganized ferments, according to Wain's book : " The Story Behind the Word " (1958).

Since 1955, it has been clearly evident that no guiding hand presided over the rapidly growing science of enzymology and only limited relationships could be claimed with cancer because of gross confusion in unit definitions, nomenclature disagreements and the multiplicity of terms employed. In 1961 international agreement was finally reached (Report of the Commission on Enzymes, I.U.B., 1961), but full international acceptance is still to come (King and Campbell, 1961 ; Freeman, 1961 ; Thompson, 1962).

The report of the Commission on Enzymes of the International Union of Biochemistry submitted and approved in 1961 after prolonged and arduous work on the part of the commission along with extensive correspondence with biochemists throughout the world, has the embodiment of its recommendations in this review. The scheme of classification of enzymes as oxidoreductases, transferases, hydrolases, lyases, isomerases and ligases serves as a reference framework and, the popular name, the new trivial name and, on occasion, an abbreviation are employed to minimize confusion.

### OXIDOREDUCTASES

Oxidoreductases, which comprise more than 25 per cent of the known enzymes, bear a positive but limited significance to cancer. Relatively few enzymes from this group are altered in cancer, but these may supplement histologic findings and may or may not indicate the organ origin of the tumor. Oxidoreductases are diagnostically non-specific in localized or disseminated neoplasia and, to variable degrees, are influenced by disease states other than malignant neoplasia. Dehydrogenases, reductases and oxidases are part of this class.

Lactic Acid Dehydrogenases (Lactate Dehydrogenases), LDH, have been very extensively studied to establish a relationship to cancer, since well known to be normal components of tissue and all body fluids, as well as being responsible for forming lactic acid in glycolysis and for oxidation of lactic acid in respiration. In short, LDH is known to occur in all known glycolyzing cells (Long, King and Sperry, 1961 ; Fishman, 1960*a*, *b*). Currently many investigators have reported on the structural differences among LDH fractions, for example, Hill (1958, 1961), Plageman *et al.* (1960), Wroblewski (1958*a*, 1959*b*), Nisselbaum and Bodansky (1961*a*, *b*), Dixon and Webb (1958), Vesell and Bearn (1958) and Winer and

Schwert (1958). Up to five iso-enzymes have been indisputably demonstrated by various electrophoretic and chromatographic technics (Sayre and Hill, 1957; Hill, 1958, 1961; Wieme, 1959*a*; Starkweather *et al.*, 1961; Vesell and Bearn, 1961; Wörner and Martin, 1961; Latner and Skillen, 1961; Dioguardi *et al.*, 1961*b*; Laursen, 1962). These findings may indicate the specific tissue from which they derive and such data would enable the clinical chemist in the future to not merely report an LDH activity in the patient but supply the cancer diagnostician with the cellular source from which originating. This kind of diagnostic potential becomes obvious since LDH is elevated in most cancer as well as many other diseases and consequently is not an adequate screening test *per se.*

LDH in abnormal levels in body fluids has been reported in relation to leukemia (Hill and Levi, 1954; Bierman *et al.*, 1957; Wroblewski *et al.*, 1957; Bodanski, 1961), gastric cancer (Schenker, 1959), lymphomas (Bierman *et al.*, 1957; Wroblewski *et al.*, 1957), central nervous system involvement by metastatic carcinoma (Wroblewski *et al.*, 1957) and in most malignant tumors (Wroblewski and LaDue, 1955; White, 1958*a*, *b*; Van Rymenant and Tagnon, 1959*a*; Fylling, 1961).

Wroblewski (1958*a*) reported several different mechanisms to explain the alterations in LDH activity in serum, serous effusion and cerebrospinal fluid. Understanding of known mechanisms contributing to LDH activity alteration in body fluids becomes necessary in order to correlate the quantitative and serial changes in LDH activity with experimental and/or clinical factors.

Hill and Levi (1954) reported the first important claim that LDH activity was elevated in individuals with neoplastic disease and inspired much research to be centered around LDH ever since.

Reviewing the observations of most investigators since 1956 indicates that several types of neoplastic diseases seem associated with high LDH levels and that the degree of elevation appears related directly to the extent of spread of the neoplastic process. In fact, the elevated amount of LDH activity seems apparently uncharacteristic of the malignant cell itself and probably a reflection of its rapid growth rate and more active metabolism. This seems the real importance of measuring LDH activity at this time.

Malic Acid Dehydrogenases (Malate Dehydrogenases), MDH, have only been investigated in body fluids to a limited extent. Kärcher (1962) reported recently in his well-documented article: "Die Bedeutung der Enzymdiagnostik in der Strahlentherapie", that the value of enzymological diagnosis during radiation therapy of patients with tumors should be stressed. He employed MDH and LDH as regulators of his radiation treatment and stated that constant control of these two enzymes in serum could give valuable clues as to the negative or positive effects of radiation therapy on the tumor.

Some attention in recent years has also been devoted to understanding and demonstrating up to three isoenzymes of MDH (Vesell and Bearn, 1958).

Isocitrate Dehydrogenases, ICD, have received the most attention of any enzyme in the citric acid cycle (Bodanski, 1961). Wolfson and Williams-Ashman (1957) developed a method for determing ICD activity and Wolfson *et al.* (1958), and Sterkel *et al.* (1958), reported that levels of this enzyme were often elevated in malignancies with metastases to the liver, but were seldom changed in portal cirrhosis and in extrahepatic obstruction of the common bile duct.

Although at this stage, ICD is not generally considered of significant value in cancer diagnosis, it possesses one outstanding asset according to Bodansky

(1961), namely : the marked rise in serum ICD and the enlargement of the liver were consistent with rapid metastatic growth of tumor in the liver during the terminal period of the patient's life.

Tyrosinase (Catechol Oxidase), the enzyme of many names ; phenolase, phenol oxidase, DOPA oxidase, catechol oxidase, potato oxidase (Long *et al.*, 1961), but assigned a new systematic name (Report of the Commission on Enzymes, I.U.B., 1961), *o*-diphenol : $O_2$ Oxidoreductase, and herein referred to by its trivial name, catechol oxidase, has been designated an enzyme under genetic control (Strauss, 1960) which contains copper. Winkelman (1961) stated that this enzyme was fulfilling a special role in juvenile melanoma conditions. Earlier, Fitzpatrick (1952) had reported that the catechol oxidase reaction appeared to be associated with the malignant character of human pigment cells, called melanogenocytes, and of the malignant neoplasms, melanoma cells alone formed melanin incubated in tyrosine, while other malignant tumors tested contained no catechol oxidase activity.

It was further shown by Fitzpatrick (1952) that nonmalignant pigment cells in normal skin required activation of the catechol oxidase system by a stimulating factor, for example, radiant energy, in order to form melanin when incubated in tyrosine. Such a catechol oxidase reaction therefore may provide a new approach to (first) the diagnosis of malignancy in pigment-cell neoplasms, for example, functional nevi versus malignant melanoma, and (second) the differentiation of amelanotic malignant melanoma from other highly undifferentiated malignant lesions which it simulates, such as fibrosarcoma, lymphoma and squamous-cell carcinoma.

Succinic Acid Dehydrogenase (Succinate Dehydrogenase), SDH, has excited much curiosity in recent years, largely because of the reported possibility by Kaufman and Hill (1960) that SDH activity in malignant cells might be significant since SDH was reduced in infected HeLa cells, a strain of human epithelium derived from carcinomatous tissue of the cervix. Even more recent, De *et al.* (1962) supplemented the information on this flavoenzyme when they reported a nucleolar localization of SDH in both normal and malignant cells from epidermoid carcinomatous tissue of human cervix, this being a new finding apart from its well established cytoplasmic counterpart, suggesting the possibility of isoenzymes existing.

Cytochrome Oxidase, CO, has remained notable for its negative activity in cancer. Hoffman *et al.* (1951) reported, as a result of cytochemical studies, that human leukemic cells and those with lymph nodes affected by Hodgkin's disease contained approximately the same degree of CO activity as their normal counterparts. Greenstein *et al.* (1944) had previously reported the same findings with human leukemia patients. Some investigation is proposed to clarify by explanation why cytochrome oxidase activity is unaffected by conditions of leukemia.

Glucose-6-Phosphate Dehydrogenase, a glycolytic enzyme, has been widely investigated by researchers in the field of intermediate metabolism because of its role as catalyst in the first step of the pentose phosphate cycle. Stave and Oehme (1961/62) have reported this enzyme significantly decreased in parablastic leukemia. Pearse (1960) has reported from his studies of growing tumors and especially in squamous carcinoma, greatly increased activity of glucose-6-phosphate dehydrogenase observed in the cells of the growing edge.

6-Phosphogluconic Dehydrogenase (Phosphogluconate Dehydrogenase) PGD, another catalyst in the pentose phosphate cycle has been only recently catapulted into importance in cancer because of the promising work of Bonham and Gibbs (1962) who have proposed and described a new enzyme test as an aid in the diagnosis of gynecological cancer in which the increased activity of PGD in vaginal fluid has significance. According to their investigation, the diagnosis of cancer appeared to be equally as effective in cases of corporeal adenocarcinoma and mesodermal tumors as with carcinoma of the cervix, including carcinoma-*in-situ*. This enzymological approach offers an alternative to the established and acknowledged important cytodiagnosis of early cancer of the uterus by providing a simpler, less expensive, but consistent biochemical screening test that may disclose more than the histologist can demonstrate regarding possible metabolic changes in cancerous and precancerous cells. Furthermore, enzymology and cytology can both afford to explore in greater detail the exfoliated cells from the female genital tract. Other than this report, predominantly negative results have been obtained by various research groups and consequently, PGD has been considered only physiologically interesting. Weber (1959) reported PGD had merit as a functional test in his study of hepatic enzymes as related to pathology of glucose-6-phosphate metabolism. Van Rymenant and Tagnon (1959*a*) reported normal values of PGD activity found in patients with carcinoma of the breast and extensive metastases as well in patients with carcinoma of the prostate.

Glyceraldehydephosphate Dehydrogenases (Triosephosphate Dehydrogenases), TPD, have become important glycolytic enzymes in recent studies. Stave and Oehme (1961/62) have reported TPD as consistently decreased in patients suffering from acute parablastic leukemia when isolated leukocytes were examined for determination of enzyme patterns, but to a lesser extent than glucose-6-phosphate dehydrogenase on the same patients.

Glutathione Reductase, GR, activity has few reports in the literature dealing with its specificity, but it seems predestined to play some role in the cancer mechanisms of the body. Manso and Wroblewski (1958) reported GR an important enzyme in oncology. They stated increased serum GR activity was detected in patients with carcinoma usually in the disseminated phase of the disease, but extention of these observations on the relationship of GR activity of serum, cerebrospinal fluid and serous effusions to cancer and various diseases would be necessary in order to evaluate the clinical significance of alterations of GR activity in body fluids. Van Rymenant and Tagnon (1959*b*) reported increased activity of GR had been observed in the serum of patients with cancer usually of the generalized type, whereas lymphomas were not accompanied by increases of GR activity in serum. They further reported that a few studies were conducted on enzyme activities in serous effusions, suggesting that the enzyme activity was increased only when malignant cells were present in the effusion.

Catalase activity studies have been carried out by numerous researchers with divergent opinions on this hemoprotein enzyme. Bergel (1961) made several references to its possible role in human cancer based on the knowledge that the catalase level decreased continuously during the growth of the tumor, unless the neoplastic growth was surgically removed when the enzyme regained, after a few days, its normal level. Such a behaviour suggested inhibitor characteristics. Feinstein and Vetter (1961) attempted to employ catalase as a cancer therapeutic agent with negative results. Adams and Berry (1956) reported that tumor growth reduced the initial liver catalase activity much more than it reduced the increased activity obtained on incubation, showing that there was a potential catalase activity in animal liver which was not detectable by estimating the level of the enzyme.

Bodansky (1961) in his discussion of serum enzymes corresponding to metabolically involved tissue enzymes cites that too few instances of human plasma catalase have been studied and, of several carcinoma cases followed enzymatically, findings for catalase activity were not elevated. Kidson (1962) reported certain variation in catalase activity in human leukocytes, finding activity of normal granulocytes higher than that of normal lymphocytes. He found levels in paramyeloblastic leukemia and chronic myeloid leukemia higher than in normal granulocytes.

Xanthine Oxidase, XO, a purine metabolizing enzyme that displays equally low activity in normal and cancerous female breast tissue, has been seriously studied since its introduction by Figge and Strong (1941) who claimed it had a role in cancer. Much data has been furnished by Bergel (1961). Skepticism and much controversy have followed the report of Figge and Strong aforementioned. General agreement existed that although XO might be related to the carcinogenic process, this enzyme could be eliminated almost completely from the liver by dietary means alone without cancer resulting. From the current literature it is not known whether the recent claims by Weber (1961), Bergel (1961) and Bennet *et al.* (1960), that tumor XO activity is slightly lower than normal is due to presence of less enzyme or, to the presence of an inhibitor of XO, or to the absence of an essential activator or auxilliary system. Further definition of this deficiency might lead to a chemotherapeutic means of overcoming the deficiency.

Tryptophan Peroxidase, TPO, has been studied rather widely by enzymologists and chemists interested in tryptophan metabolism (Posnanskaya, 1958 ; Douglas, 1959, unpublished results ; Chan *et al.*, 1960 ; Long *et al.*, 1961 ; Bergel, 1961). TPO of the liver is considered an adaptive enzyme controlling tryptophan metabolism and theoretically with such a relationship to an essential amino acid, an understanding of its role in disease is necessary. Evidence presented by Posnanskaya (1958) suggests that the adaptive increase of TPO system of the liver may not be associated with *de novo* synthesis of protein. The peroxidase reaction is used in several forms to attempt differentiation between the later " blast " forms of lymphoblasts and myeloblasts, particularly in acute leukemia. Peroxidase-positive granules may be present in the cytoplasm of blast cells of the granulocyte series and very early pre-myelocytes before ordinary granules are visible and they are never present in lymphoblasts. Such a laboratory method is of limited value and should be left to the discretion of the pathologist. There are also TPO tests for differentiating early white blood cell precursors in defining leukemoid reactions. D-Aminoacid Oxidase, a flavoprotein, is an oxidoreductase that may emerge as a vital enzyme in understanding cancer but it is still physiologically unclarified (Long *et al.*, 1961) but thought to behave as an apoenzyme (Bergel, 1961).

Bover and Candela (1961) and Candela and Bover (1961) cytochemically studied dehydrogenase activity of leukocytes and thrombocytes in blood of normal subjects affected with chronic myeloid leukemia and those with polycythemia vera, finding that an increase in the endogenous reductase activity (i.e. activity due to unspecific dehydrogenase) of the polynuclear leukocytes occurred in the cases of myeloid irritation and that a decrease occurred in the case of chronic myeloid leukemia. These findings paralleled changes observed in alkaline phosphatase activity.

Other recent contributions have been made which deserve mention in the review of cancer enzymology as related to oxidoreductases: Bergel (1960), Beck (1958), Blanchaer, *et al.* (1958), Bodansky (1956, 1958, 1959), De Lamirande *et al.* (1958), Dalgliesh (1952), Elliot and Wilkinson (1961), Frei *et al.* (1961), Green (1958), Grönvall (1961), Hayaishi (1962), Hill *et al.* (1956), Hill and Jordan (1957), Holmberg and Laurell (1951), Hsieh *et al.* (1955, 1956), Hsieh and Blumenthal (1956), Humoller *et al.* (1958), Jagt and Larsen (1960), King (1957), Kirkeby and Prydz (1959), Laursen (1959, 1962), Meister (1950), Plummer and Wilkinson (1962), Rapp and Bell (1961), Riley and Wroblewski (1960), Robert and Van Rymenant (1961), Singer and Kearney (1957), Stekol (1959), Vetter (1961), Wieme (1959*b*), Zucker and Borelli (1958), and Wroblewski (1958).

TRANSFERASES

In this category may be listed many enzymes that have been mentioned in relation to cancer, but always as non-specific enzymes for neoplastic disease and usually affected by many other disease conditions.

Glutamic Oxaloacetic Transaminase (Aspartate Aminotransferase), GO-T, and Glutamic Pyruvic Transaminase (Alanine Aminotransferase), GP-T, are most prominent in this class. Elevations in GO-T and GP-T activity levels have been associated with a multiplicity of diseases (Wroblewski, 1959*a*), but may have diagnostic importance when carefully correlated with clinical facts. Examples are metastatic and primary hepatic carcinoma which become reflections of the enzyme changes at the intracellular tissues.

GO-T and GP-T serum levels are always increased in diseases involving destruction of liver cells. Transamination itself, the reaction between an alpha-amino acid and an alpha-keto acid through which the amino group is transferred from the former to the latter, has a tendency to relate tumors to protein biosynthesis and degradation if the concept that apoenzymes are products of certain polypeptides which are products of ribonucleic acid and nucleotides. Extensive biochemical studies of enzymatic transamination have overshadowed clinical implications of GO-T and GP-T activity in humans, yet these transaminases are readily measurable by comparatively simple techniques (Karman *et al.*, 1955; Henley and Pollard, 1955; Laursen and Espersen, 1959; Laursen and From-Hansen, 1958; Wroblewski, 1958*b*, 1959*a*; Aspen and Meister, 1958). Transaminases appear to be measures of cell damage rather than cell function and can be regarded as normal intracellular enzymes. Eastham (1960) stated that when patients have infiltration of the liver, cases of carcinoma, leukemia and lymphoma may show an increased activity.

In more recent years, other transferases have received much attention but so far not achieved major significance. Phosphoglucomutase, PGM, the glycolytic enzyme (Colowick and Kaplan, 1955) that converts glucose-l-phosphate to glucose-6-phosphate and requires glucose-1, 6-diphosphate as cofactor (Fishman, 1960*b*), a reversible conversion, has been reported as elevated in patients with metastatic disease (Bodansky, 1961) and bears a consistent abnormal pattern in most cancer conditions. Bodansky (1957*a*) has developed a method for determination of PGM activity in serum and has applied it successfully to the clinical investigation of cancer of the breast. PGM occurs in all cells (Fishman, 1960*b* ; Long *et al.*, 1961) and Nigam *et al.* (1962) have reported on limiting enzyme factors for glycogen storage in tumors and shown the lesion was a defective system for glycogen synthesis owing to low activities of the enzymes involved in the synthetic process (PGM and glycogen synthetase) which were unable to accomplish efficient transformation and catalytic action in the presence of competing high rates of tumor glycolysis and normal polysaccharide degradation by phosphorylase.

Hexokinase, HK, an enzyme transferring phosphorus-containing groups and catalyzing the phosphorylation of glucose to form hexosemonophosphate, has been reported significant in leukemia. Stave and Oehme (1961/62) have demonstrated decreased activity of HK to 50 per cent of normal in parablastic leukemia. Beck (1958) also has reported HK deficiencies in leukemic cells, pointing out that the phosphogluconate oxidation pathway occurs in leukocytes, though less than 10 per cent of utilized glucose traverses this pathway and this percentage being higher in leukemic cells than in normal cells. The extent of alternative pathway metabolism is shown to be under direct control of the glucose-6-phosphate concentration and indirect control of the hexokinase level. Beck states the higher percentage of phosphogluconate pathway metabolism in leukemic cells can be attributed to their deficiency of hexokinase.

Pyruvate Kinase, PK, an enzyme catalyzing an essential step in the glycolytic or fermentative breakdown of carbohydrates (Long *et al.*, 1961) has so far very limited application in clinical medicine and based on the current literature (Bodansky, 1961), because much attention has been directed toward the investigation of PK in blood, it is noteworthy to say that this enzyme remains insignificantly altered in advanced cancer and widespread metastases.

Ornithine Carbamoyltransferase, OCT, an enzyme incompletely studied, but theoretically looming as a diagnostic potential of value in neoplastic disease of liver and other organs, remains to be developed. Reichard (1957) has reported a dependable laboratory method for the determination of OCT by a microdiffusion technique.

Phosphofructokinase, PFK, an enzyme related to KH, was studied by Neufach and Melnikova (1958) who reported considerable data to support the theory that while the rate of glycolysis in skeletal muscle does rely upon PFK power, it is really a function of HK in erythrocytes and tumors.

Phosphoribokinase, PRK, another enzyme related to HK, was introduced in 1953 by Scarano and considered to be altered in tumor growth due to direct or indirect involvement of PRK in formation of adenosine monophosphate in animal tissue from adenine (Saffran and Scarano, 1953).

Ribonuclease, RN, the enzyme that catalyzes the hydrolysis of ribonucleic acid, figures only indirectly in cancer, but much speculation has been reported over the past ten years on this physiologically important nucleolytic enzyme (Greenstein, 1954 ; Allard *et al.*, 1957 ; Houck, 1958 ; Dixon and Webb, 1958 ; De Lamirande *et al.*, 1958 ; De Lamirande and Allard, 1959 ; Fishman, 1960*b* ; Long *et al.*, 1961 ; Josefsson and Lagerstedt, 1962) and it has even been reported in recent literature that there exist several isoenzymes of RN which differ in their pH optima, their tissue origin or their nucleotide end-products (Bergel, 1961).

Galactose-1-Phosphate Uridylyltransferase, an enzyme possibly under genetic control, has been proposed as indirectly having a role in the mechanisms of cancer enzymology (Strauss, 1960).

Some relationship has been suggested between tumor development and enzymes of the fatty acid metabolism but so far is too purely chemical to interest becologists (Stern *et al.*, 1956*a*, *b* ; Robinson *et al.*, 1957). Most transferases ionng intracellular, exert minimum diagnostic value for the clinician.

HYDROLASES

In this very large class of enzymes a positive relationship has been displayed in cancer by some hydrolases, whereas other incompletely documented hydrolases appear insecurely linked to the field of cancer. So much has been written about certain enzymes in this grouping, that only the pertinent data and current reports need be reviewed.

Acid Phosphatase, ACP, an orthophosphoric monoester phosphohydrolase, an enzyme that liberates inorganic phosphate from phosphoric esters with an optimum pH of 5·4, is almost synonymous with the prostate gland. An impressive and lengthy list of standard literature has accumulated over several decades (Kutscher and Wolbergs, 1935 ; Gutman and Gutman, 1938 ; Gutman, Gutman and Robinson, 1940 ; Huggins and Hodges, 1941 ; Gomori, 1941 ; Abul-Fadl and King, 1948 ; Herbert, 1946 ; Woodard, 1952, 1959 ; Powell and Smith, 1954 ; Bonner, Homburger and Fishman, 1954 ; Bodansky, 1954*a*, 1959, 1961 ; Nylander, 1955 ; Mather, Richmond and Sprunt, 1956 ; Fishman and Davidson, 1957 ; Fishman, 1960*a* ; Pearse, 1960 ; Benson, 1957 ; Bergel, 1961 ; Annino, 1960 ; Zucker and Borelli, 1959 ; Long, King and Sperry, 1961 ; Meijer, 1962). It is redundant to say this enzyme is extremely important in the diagnosis of metastasizing carcinoma of the prostate. Gutman and Gutman (1940) having reported from their data that increases to several hundredfold over the normal range for ACP activity were noticeable in subjects with prostatic carcinoma especially when the tumor extended outside the gland. ACP estimations are generally made either by the quantity of phenol liberated from sodium phenylphosphate, as in the King-Armstrong method, or by the liberated phosphate from glycerophosphate, as in the Bodansky method. Fishman and Lerner reported in 1953 their well known method for " prostatic " acid phosphatases which gave excellent correlation with the presence of proven cancer of the prostate (Fishman, Bonner and Homburger, 1956 ; Fishman *et al.*, 1953). Fishman *et al.* (1953) reported evidence that, by means of an l-tartrate inhibitor, one could measure largely, but not exclusively, prostatic acid phosphatases based on both experimental and clinical data. In the numerous attempts to render the ACP determinations more specific for prostatic origin, the works of many investigators are noteworthy. Reynolds *et al.* (1956) used copper to inhibit erythrocyte ACP and reported a large percentage of patients with widespread cancers of the female breast or of the prostate gland had significantly elevated values for copper resistant ACP. Abul-Fadl and King in 1948 employed a simple formaldehyde treatment to distinguish between the high ACP of prostatic origin and those accompanying other conditions. while earlier Herbert in 1946 had reported employing an alcohol incubation treatment for the same purpose. Gray (1959) reported that the plasma acid phosphatase derived from the prostate was inactivated by treatment with alcohol and this behavior useful in deciding whether an increased ACP activity level was due to prostatic carcinoma or to liberation of the ACP from red blood cells by hemolysis. Woodard (1959) reported it possible to distinguish rather clearly between the level of the prostate and that of the erythrocytes, but not so simple to distinguish between prostatic ACP and acid phosphate from other sources. Zucker and Borelli (1958) reported normal serum acid glycerophosphatase appeared to come from blood platelets. In contrast to normal individuals, acid glycerophosphatase activity was found in serum from platelet-poor plasma obtained from patients with metastatic carcinoma of the prostate, suggesting that a more sensitive test for pathological elevation of this enzyme activity in prostate cancer was provided when the contribution of ACP from the platelets was avoided. Methodology has undergone critical examination over the years and the results render accurate and sensitive data.

Regarding the possible isoenzymes of ACP, Meijer in 1962 reported three different non-specific fractions characterized by different pH optima between 3·4 and 5·8 in the liver and spleen after intraperitoneal administration of macromolecular substances as Dextran and Polyvinylpyrrolidone, which caused the entire enzyme complex to rise. Meijer also noted the increase in activity was not equally distributed.

Part of the clinical value of this enzyme is not only to detect the occurrence of metastases in this prostatic disease, but to assess the progress of therapy in such cases (Harper, 1958).

Much work and study have gone into the relationship of ACP to mammary cancer (Reynolds, *et al.*, 1956 ; Lemon *et al.*, 1958 ; King, 1959 ; Eastham, 1960 ; Fishman, 1960*b*) with moderately positive, but questionable findings. Reynolds, Lemon and Byrnes (1956), then Lemon, Reynolds and Kelley (1958) claimed when using copper-resistant ACP that 74 per cent of a group of female patients with mammary metastatic carcinoma had elevated enzyme levels. Lemon and Wisseman (1949) by employing histochemical and quantitative microchemical methods reported carcinoma of the breast, bronchus, skin, bladder and gastro-intestinal tract were richer in ACP than the tissue of origin. Such reports were in agreement with earlier histochemical studies on human tumors by Gomori (1941). Hudson *et al.* (1955) demonstrated that liver appeared to be implicated in the metabolism of serum ACP of prostate origin and found values grossly elevated (over 1000 Gutman units per 100 ml. plasma) in cases with metastases of the liver from prostatic cancer. Burstone (1958) demonstrated that lung carcinoma showed, by an azo-dye histochemical staining technic for ACP, groups and masses of intensely-staining neoplastic cells and indicated that this common characteristic of lung tumors might be a useful tool in studying bronchial aspirations.

Adventitious elevations of ACP have appeared frequently in the literature (Hock and Tessier, 1949 ; Woodard, 1952 ; Fishman *et al.*, 1953 ; Bonner *et al.*, 1954 ; Dybkær and Jensen, 1958 ; Bodansky, 1961 ; Ladehoff and Rasmussen, 1961). It has been noted that massage, palpation, trauma, rectal examination or any pressure exerted on the prostate may result in sudden elevation of the ACP activity level. This occurrence seems best comprehensively supported by the theory of Ladehoff and Rasmussen (1961) who stated that very likely the increased fibrinolysis in blood observed in transvesical prostatectomy was caused by a release of prostatic tissue activator into circulating blood during the manipulations of enucleation together with damage of and adsorption from the particularly active " surgical capsule ", which was the site of cleavage.

Alkaline Phosphatases, ALP, have been so completely reviewed in the literature by Bodansky (1961) and supplemented to such an extent (Franseen and McLean, 1935; Gutman *et al.*, 1936; Bruns and Jacob, 1954; Bodansky, 1956, 1959; Schlamowitz and Bodansky, 1959; Gutman, 1959; Gray, 1959; Fishman, 1960*a*; Fischer and Siebert, 1961; Barnes and Cope, 1961) that condensation of all published reports would be difficult. ALP activity levels are commonly elevated in osteogenic sarcoma, in metastatic carcinoma to bones resulting in osteoplastic changes, in myelogenous leukemia, in carcinoma of the pancreas, in hyperparathyroidism accompanying cancer of the parathyroid glands and in hepatic disorders such as secondary carcinoma. Iwatsuru and Nanjo (1939) reported elevated ALP activity in blood of a chronic myeloid leukemia patient. Iwatsuru and Minami (1934) reported normal values for ALP in acute lymphatic leukemia. Xefteris *et al.* (1961) reported the prognostic significance of enzyme levels in the remissions of chronic granulocytic leukemia patients treated with Busulfan (Myleran) therapy, presenting data suggesting the return to normality of ALP activity might presage a lengthy remission. This parameter was based on twelve cases. However, King (1957) stated no useful correlation of ALP in leukocytes was possible with cancer.

A contribution to the study of serum ALP and its role in cancer has been made by Chevillard in 1945, who demonstrated platelet phosphatase entering the serum during clotting, this source being more extensively elaborated upon in more recent work of Zucker and Borelli (1958, 1959). Chevillard's report stimulated many other investigators apparently, because other important reports were published subsequently by Wachstein in 1946 and Valentine in 1956 on the role of ALP in blood. Before 1945 principal knowledge of leukocyte ALP activity was attributed to Kay who introduced same in 1930 and the work of Roche (1931), then Fiessinger and Boyer (1935).

Gutman (1959) pointed out the limitations in presently considering serum ALP determinations in the differential diagnosis of hepatobiliary diseases; referring, among other references, to the failure to distinguish obstruction of the intrahepatic from that of the extrahepatic biliary tract, and the failure to differentiate benign from malignant occlusion of the extrahepatic biliary tract. Regardless, alkaline phosphatase activity determinations are the most sensitive available chemical criterion of extra-, or intrahepatic biliary tract disorders.

Kerppola (1951) reported no simple correlation has been shown to exist between tumor growth and the concentration of phosphatase in ordinary tissue; and, as regards the origin of the phosphatase in the blood cells, it was of interest that no correlation was observed in cases between serum phosphatases and the blood cell phosphatase. Kerppola further reported in detail on the presence of particular phosphatases related to cellular reaction, claiming that some phosphatase might destroy cells or their nuclei, that some phosphatase activity was purely physiological, that some phosphatase concentration was related to accelerated or malignant growth of bone marrow of tissue associated with bone formation; and that his observations disclosed many unexplained appearances of phosphatase activity in blood and bone marrow cells.

Gutman (1959) emphasized the distinct value of an increased ALP activity in skeletal diseases and neoplasia involving bone. Fishman *et al.* (1953) asserted that because the human prostate produces an alkaline phosphatase which is capable of being inhibited by tartrate in contrast to the enzyme in erythrocytes, this difference could be used for diagnostic purposes in cases of prostate cancer.

Decreasing values in serum ALP activity can be interpreted as enzymological evidence of relapsing disease (Myers and Bodansky, 1957) and such has been also described by Griboff *et al.* (1954), as heralding the onset of hypercalcemia in carcinoma of the breast which in turn may indicate exacerbation of the disease.

Keiding in 1959 reported three different ALP activities in serum by zone electrophoresis technics in his studies on cancer patients although from a heterogenous group. Although no more than three types of ALP activities have been observed by any one method to date, whether starch granule electrophoresis or cellulose chromatography or immunological systems, Boyer (1961) has recently reported that at least 16 bands of non-specific ALP activities were evident in human sera following electrophoresis procedures on hydrolyzed starch gel, but not all occurred in a single individual. Moss in 1962, using butanol-extracted liver preparations, showed variations in the pattern of ALP isoenzymes, probably due to methodology. Hodson *et al.* in 1962, continuing their previously reported isoenzyme work (Hodson *et al.*, 1961), claimed tissue-specific alkaline phosphatase isoenzymes were found. It is necessary for additional research along these lines to show the relationship of these ALP components in differential diagnosis of cancer.

Much speculation has centered around another phosphatase (Long *et al.*, 1961), 5-Nucleotidase, 5NT, which is optimally active at an alkaline pH and has an alteration in many disease states (Bergel, 1961 ; Bodansky, 1961 ; Young, 1958 ; Van Rymenant and Tagnon, 1959*b*). Dixon and Purdom (1954) reported 5NT activity level low in osteogenic tumors, breast cancer and spinal neoplasms. Van Rymenant and Tagnon (1959*b*) stated the diagnostic value of 5NT determination could be advantageous in comparison to ALP because it was uninfluenced by bone disease. 5NT determinations may prove to be of clinical value in view of the evidence that it is at least as sensitive as the serum ALP in detecting the presence of biliary tract obstructions and is more selective because values are not increased in disease of bone associated with increased osteoblastic activity.

Beta-Glucuronidase is another important hydrolase that has undergone much scrutiny by investigators (Long *et al.*, 1961) and occurs in a wide variety of tissues with high concentrations found in liver, spleen and endocrine tissues (Fishman, 1960*b*). Thorough assay methods have been developed (Colowick and Kaplan, 1955 ; Fishman *et al.*, 1948 ; Fishman *et al.*, 1947) and histochemical procedures (Seligman, Nachlas, Manheimer, Friedman and Wolf, 1949) have demonstrated both animal and human tumors rich in this enzyme. It has been reported that the buffy coat of blood has high beta-glucuronidase activity (Fishman, 1960*a*) ; and, Anlyan *et al.* (1950), have studied and reported a wide range of enzyme activity in various types of leukemia with abnormally high values occurring in myelogenous leukemia based on their patients with leukemia and Hodgkin's disease. Fishman *et al.* (1947) found strikingly elevated values in many human neoplasms and above normal values in the vaginal fluid of a large percentage of women with cancer of the uterine cervix (Fishman *et al.*, 1954). Beta-glucuronidases are elevated in primary neoplasms of the breast, uterus, ovary, stomach colon and in their metastases to other organs and lymph nodes. There is a correlation between the beta-glucuronidase content of vaginal fluid and of cervical cancer under certain conditions and between the activity of this enzyme in exudates and the presence of cancer (Homburger and Fishman, 1956). Elevations in cases of cancer of the breast are common (Cohen and Huseby, 1951*a*, *b* ; Fishman, 1960*b* ; Fishman *et al.*, 1954). Much research has been performed and will be continued towards understanding this hydrolase (Fishman, 1949 ; Fishman and Anlyan, 1947 ; Fishman *et al.*, 1959 ; Fishman and Bigelow, 1950 ; Homburger, 1960 ; Fishman and Mitchell, 1959 ; Bodansky, 1961 ; Boyland *et al.*, 1957 ; Rauramo, 1959*a*, *b* ; Odell *et al.*, 1949 ; Kasdon *et al.*, 1950 ; Kasdon *et al.*, 1953).

There are numerous other hydrolases which have so far an insignificant role in general cancer but may figure more prominently in the future ; as, Arylsulphatase (Dzialoszynski, 1957 ; Dodgson and Spencer, 1957 ; Dodgson *et al.*, 1956 ; Tanaka *et al.*, 1962), Amylases (Ende, 1961 ; Best and Taylor, 1961 ; Hansen and Jacobsson, 1952 ; Jacobsson and Hansen, 1952 ; Jørgensen and Svendsen, 1961), Adenosine Deaminase (Schwartz and Bodansky, 1959 ; Stekol, 1958 ; Straub *et al.*, 1957), Glucose-6-Phosphatase (Weber, 1959 ; Weber and Cantero, 1960 ; Kit, 1960), Arginase (Forsell and Palva, 1961 ; Friedman and Becker, 1955 ; Pravdeena, 1957 ; Roberts, 1948), Plasmin or Fibrinolysin (Long *et al.*, 1961 ; Astrup, 1956), Elastase (Lewis *et al.*, 1956), Chymotrypsin (Lundh, 1957 ; Billow *et al.*, 1960 ; Brandborg *et al.*, 1961), Trypsin (Lundh, 1957 ; Astrup and Albrechtsen, 1957 ; Astrup and Sterndorff, 1955 ; Nardi and Lees, 1958), Pepsin or Uropepsin (Gray *et al.*, 1955 ; Jørgensen, 1954 ; Bock, 1954 ; Christensen, 1957*a*), Deoxyribonuclease (Kowlessar *et al.*, 1953 ; Brody, 1958 ; Brody and Balis, 1958), Leucine Aminopeptidase (Dioguardi *et al.*, 1961*a* ; Goldberg and Rutenburg, 1958), Cholinesterase (Winkelman, 1961 ; Gal and Roth, 1957 ; Torp, 1956 ; Stovner, 1955 ; Sabine, 1951 ; Augustinsson, 1955), Esterases (Green and Jenkinson, 1934), Cathepsins (Libenson and Jena, 1957), and the Lipases (Cohn and Kaplan, 1960 ; Borgström, 1957 ; Bergel, 1961 ; Best and Taylor, 1961).

### LYASES, ISOMERASES AND LIGASES

Relatively few lyases bear significance to date. Most noteworthy are aldolase, hyaluronidase, carbonic anhydrase, tryptophan synthase, hydroxytryptophan decarboxylase and possibly ketotetrosealdolase. The latter enzyme has been confused biochemically with aldolase (Wolf, Forster and Leuthardt, 1957) and is only negatively important in that it is consistently absent from serum in instances of liver metastases and normal serum, although appearing in serum of patients with viral hepatitis and other diseases. Hydroxytryptophan Decarboxylase has been examined by many protein and enzyme researchers (Cantero, 1955 ; Douglas, 1959, unpublished results ; Kizer and Chan, 1961 ; Kizer, 1962) who have related it to the minor pathway of tryptophan metabolism and correlated such activity with cancer susceptibility, not yet successfully. Along the same line of investigation, studies have embraced tryptophan synthase (Smith and Yanofsky, 1962). Summarily, from the bulk of work done of hyaluronidase, no true correlation occurs between hyaluronidase content and the type of tumor (Dux, Guerin and LaCour, 1948) nor from other correlations speculated upon (Truedsson, 1951 ; Bolio-Cicero *et al.*, 1961 ; Gaines, 1960 ; Winslow and Taylor, 1960 ; Ekman *et al.*, 1953 ; Faber and Schmith, 1950*a*, *b* ; Winslow and Enzinger, 1960). Aldolase is a non-specific enzyme, elevated in most cancer conditions, but also in many other diseases. Baker and Govan (1953) found aldolase activity consistently high in the serum of patients with advanced carcinoma of the prostate and expressed the opinion that this enzyme was a better index than acid phosphatase for the evaluation of the status of the patient. Much earlier work was done by Sibley and Lehninger (1949) and recent reports have been made by White (1958*a*, *c*) and Løken (1956). The full diagnostic value remains unestablished (White, 1958*b*). Some new lyases are insufficiently studied to report (Brüggemann *et al.*, 1962 ; Brüggemann and Waldschmidt, 1962).

Isomerases, those enzymes in muscle and other tissue that catalyze the conversion of glucose-6-phosphate to fructose-6-phosphate, a reaction of importance in glycolysis, are indefinitely established in cancer enzymology. Two isomerases are outstanding at this stage and both could be classified by the organic chemist as intramolecular oxidoreductases interconverting aldoses and ketoses.

Triosephosphate Isomerase, TPI, has been measured in serum (Colowick and Kaplan, 1955) ; and, in cases of generalized cancer was positively elevated in hepatic metastases and occasionally constituted the sole enzyme change in the serum (Robert *et al.*, 1961). Robert *et al.*, 1961, noted TPI activity level elevated in cases of leukemia ; but, demonstrated that the value of this determination in differential diagnosis seemed restricted to the detection of liver metastases in patients with cancer (Van Rymenant and Robert, 1961).

Phosphohexose Isomerase (Glucosephosphate Isomerase), GPI, has been studied extensively by Bodansky (1954*b*, *c*, 1955, 1956) and shown to be raised in patients with metastatic cancer of breast or prostate and even more interesting, shown to have an " isomerase-mutase activity ratio " (Bodansky, 1957*b*) in serum, this proposed as an index of metastatic growth in the skeleton and/or liver within certain limitations. The ratio aforementioned was the result of Bodansky's comparison studies with serum phosphoglucomutase (1957*a*) and GPI (1957*b*) in metastatic cancer. An impressive degree of correlation was reported (Bodansky, 1954*c*) to exist between increases in serum GPI activity and growth of metastatic tumor in bone, as judged clinically, and by biochemical methods, particularly by the urinary calcium excretion. Bodansky has demonstrated that such GPI activity can provide a useful index to growth or regression of tumor. Decreases toward normal GPI levels were associated with evidence of tumor regression whereas marked and sustained elevations very often preceded widespread metastases and death. but increases in GPI activity after a sequence of repeatedly normal levels was frequently the first herald of renewed growth of tumor. Strangely, much of the enthusiasm surrounding this enzyme has subsided in the past few years.

Israels and Delory (1956) have reported GPI significant in leukemia and, according to Van Rymenant and Tagnon (1959*a*), the GPI activity of red blood cells is 160 times greater than that of plasma ; however, in many types of hemolytic diseases, the GPI activity of plasma was low and contrasting with a high level of LDH activity, thus creating an increased ratio of LDH to GPI which might be indicative of the existence of a hemolytic process in the blood of the patient (Blanchaer *et al.*, 1958).

Myers and Bodansky (1957) reported a study of a patient with metastatic cancer of the breast in which two parameters of tumor activity, serum GPI and urinary-calcium, were determined over a nine-month period. They obtained high enzyme activity which possibly reflected active soft tissue disease in the liver although there was no biopsy proof of liver metastases.

White (1958*b*) concluded that abnormal serum GPI activity might be a result of, and also an indicator of muscle wasting in many patients with cancer.

Ligases, perhaps better known in enzymology as Synthetases, those enzymes which catalyze the joining together of two molecules coupled with the breakdown of a pyrophosphate bond in adenosine triphosphate (ATP) or a similar triphosphate (Report of the Commission on Enzymes, I.U.B., 1961), are as a whole unfamiliar to the cancer enzymologist.

### INFLUENCING FACTORS ON ENZYME ACTIVITIES AND DISCUSSION OF CARCINOCHEMOTHERAPY

When the work of Braunstein and his colleagues (1958, 1962, 1962*a*, 1962*b*) who have given such detailed reports on their histochemical studies of enzymatic activity of lymph nodes and found it possible to identify cells by their enzyme content as well as further substantiating the classification of malignant lymphoma based upon conventional staining technic ; and the work of Brody and his colleagues (Brody, 1958 ; Brody and Balis, 1958) who have shown completely different paterns of ribonuclease and deoxyribonuclease activities in normal and neoplastic growth as they demonstrated a positive relationship between the growth rate of tissue and their RN and deoxyribonyclease activities ; and the works of Allard, De Lamirande and Cantero (Allard, De Lamirande and Cantero, 1957 ; Cantero, 1955 ; De Lamirande, Allard and Cantero, 1958 ; De Lamirande and Allard, 1959) who have reported the results of extensive explorations of intracellular distribution of various enzymes in primary and transplantable liver tumors and compared those results with those of azo-dye fed rats and regenerating liver and noted such enzymes as ACP, ALP, RN, cathepsin, adenosine triphosphatase and glutamic dehydrogenase disclosed a distinct variation of enzyme pattern in normal and tumor conditions ; and when other important works reported by Bergel (1956, 1961), Vetter and Griesche (1961), Vetter (1961), Morton (1958) and many more are reviewed, it then becomes a postulate that human and animal enzyme activities in the body are altered in cancer. Specific tumors have produced certain enzymes ; as osteogenic sarcoma has produced ALP, or specific organs have secreted, when they are the site of cancer, certain enzymes as carcinoma of the prostate has secreted ACP, or general or localized reactions accompanying certain cancers have produced LDH which correlated with the state and severity of leukemia (Homburger, 1960 ; Bierman *et al.*, 1955). The level of activity of beta-glucuronidase in the vaginal fluid is increased in nearly every case of cancer of the cervix (Fishman *et al.*, 1954 ; Kasdon *et al.*, 1953). The level of activity of uropepsin in benign gastric ulcer is high but in 80 per cent of gastric cancers is low (Gray *et al.*, 1955). The presence of tumor and distribution of enzymes are related.

The other factors, chemical and physiological and biophysical, are concisely stated in textbooks, reference books and various periodicals (Fenton, 1960 ; Ruch and Fulton, 1960 ; Long *et al.*, 1961 ; Wolfson *et al.*, 1958 ; Wroblewski, 1959*a* ; Wroblewski *et al.*, 1958 ; Robins, 1957 ; Endahl and Kochakian, 1957 ; Plummer and Wilkinson, 1962 ; Mason, 1958 ; Norberg, 1961 ; Mendelsohn and Bodansky, 1952 ; Fishman and Davidson, 1957) in considering factors influencing enzyme activity for possible cancer applications.

Hardly any publication on animal studies reporting enzyme activities in numerous hepatomas, lymphomas, mammary tumors, rhabdomyosarcomas and adenocarcinomas can provide more extensive background for the enzymic characteristics of tumors as contrasted to normal tissues than the reports of Greenstein (1954). Similar systematic studies with human tumor material have yet to be done due to difficulties in collecting specimens of human cancer According to present status, much more must be understood regarding influencing factors on enzymes.

Fishman (1960*a*) stated control of tissue enzyme activity may be genetic, dietary, hormonal, sexual and substrate adaptation.

There is some difficulty encountered in discussing influences upon enzyme activity due to variable usage of certain words. " Impaired " as used by Warburg (1956), for an example of confusion, was defined as involving any combination of the following : *a*. high rate of glycolysis to respiration ; *b*. low absolute value for oxygen consumption ; *c*. inefficient or uncoupled respiration ; and, *d*. low succinate oxidative response. Potter (1958) and Weinhouse (1960) had different definitions. Interpretations of cancer enzymology were handicapped because of a lack of exact definition. The summary of Fishman (1960*a*) presented an excellent brief account of all the various aspects of tumor enzymology.

Methodology for following these enzymes referenced and their influencing factors are amply covered by many enzymologically oriented scientists (Colowick and Kaplan, 1955, 1962 ; Glick, 1959 ; Annino, 1960 ; Aspen and Meister, 1958 ; Astrup and Albrechtsen, 1957 ; Augustinsson, 1955 ; Baker and Pellegrino, 1954 ; Karmen *et al.*, 1955 ; Natelson, 1961 ; Seligson, 1961 ; Josefsson and Lagerstedt, 1962 ; Schlamowitz and Bodansky, 1959 ; Seligman *et al.*, 1949 ; Powell and Smith, 1954 ; Thiers and Vallee, 1958 ; Fraenkel-Conrat, 1957 ; Schmith and Faber, 1950 ; Tammelin, 1953, Udenfriend *et al.*, 1958 ; Cohn and Kaplan, 1960 ; Christensen, 1957*a*, 1957*b* ; Dawson *et al.*, 1959 ; Dean and Woodard, 1947 ; Desnuelle, 1961) impossible to be fully listed. There appear a continuous stream of new technics superceding, supplementing, complementing and modifying stock procedures so that enzyme study is fully devoid of stagnancy.

Based on the assumption that we have enzyme patterns, it is also simple to conclude that we have changes during carcinogenesis and/or the metabolism of carcinogenic agents. Under this heading, much experimentation has been and continues to be done by Boyland and his associates (Boyland and Watson, 1956 ; Boyland *et al.*, 1957 ; Boyland, 1958) with tryptophan metabolites, and many recent reports pointed out by Berenblum (1956) and Bergel (1956, 1960, 1961). Unfortunately, there exists a paucity of data dealing with the distribution and concentration of enzymes in human tumor tissue. Collaboration between enzymologists, pathologists and clinicians are occurring now and the consequences are noticeable by a new flow of valuable information.

Cancer chemotherapy includes radioactive isotopes, sex hormones, adrenal steroids, antimetabolites, non-therapeutic toxic compounds, polyfunctional alkylating agents and miscellaneous compounds. Drugs in clinical use, or under investigation, have been generally of limited value. Most investigations from a chemotherapeutic point of view have been performed on body organs not essential to life as prostate and mammary glands, thyroid glands, gonads, uterus and cervix. In the search for specific agents to counteract cancer tissue, enzymes have not fulfilled expectations to assist therapeutically.

In the comprehensive review of clinical cancer chemotherapy by Wright (1961) enzymatic therapy was conspicuously absent, and only references made to excessive phosphatase activity found in cyclophosphamide therapy ; and, altered phosphatase values found in androgen control therapy or upon administration of testosterone. Welch (1961) mentioned specifically the need to exploit the enzymic deficiencies of tumors and gave several metabolic approaches to cancer chemotherapy. Bergel (1961) added further data on enzyme therapy as he pointed out therapeutic possibilities of xanthine oxidase, ribonuclease, deoxyribonuclease, lipase and cysteine desulfhydrase. Coordinated studies of chemotherapeutic compounds and enzyme systems have produced much more rewarding results, such as the report of Reichard *et al.* (1962), which demonstrated that certain enzyme assays will benefit the following of clinical course in chemotherapy, as in 5-Fluorocil treatment, in which they found significance in enzymes of the uracil pathway during development of resistance. Cohen and Huseby (1951*a*, *b*) have noted estrogen therapy caused a marked rise in the beta-glucuronidase activity in the sera of patients being treated for mammary cancer with maximum values twice as high as the pre-therapy level. Farber (1955) and Bergel (1960, 1961) stated that when there was an indicated permanent insufficiency of enzymes, or their co-factors could be given restitution or replacement for normal status, the need was to provide a holo-, apo-, coenzyme or model system with a specific enzymic activity.

Streptokinase or plasminokinase (Dixon and Webb, 1958), the activator that converts plasminogen of blood plasma (Long *et al.*, 1961), an enzyme precursor, into a proteolytic hydrolase, called plasmin, causing lysis of fibrin and liquifaction of any fibrinous exudate, has been employed therapeutically in man to disperse such exudates (Bridgwater and Necheles, 1957 ; Fletcher, 1954). Streptokinase is generally considered a product of hemolytic streptococcal metabolism, not an enzyme. Plasmin, formerly known as fibrinolysin, has been used alone and combined with deoxyribonuclease* (Margulis *et al.*, 1961) after much basic development by Astrup *et al.* (Astrup, 1956 ; Astrup and Sterndorff, 1955 ; Astrup and Albrechtsen, 1957), Christensen (1957*b*), Bridgwater and Necheles (1957) and Tillett *et al.* (Tillett and Garner, 1933 ; Tillett, Johnson and McCarty, 1955). Deoxyribonuclease of bacterial origin has also been employed clinically in the treatment of purulent exudates (Bergel, 1961 ; Tillett, Johnson and McCarty, 1955). Chymotrypsin†, a mixture of pancreatic enzymes (Cigarroa, 1960), was employed to minimize the edema that followed radical mastectomy and neck dissection for malignant tumor. Topical applications of proteolytic enzymes augment the bactericidal properties of the antibiotics in dermatological cases, performing only a palliative function. With the medical trend to employ multiple aids for a common purpose, and the fact that proteolytic enzymes achieve no cure alone ; and certainly, no reliable practical experiences in cancer therapy have been documented, it is better to refer to certain enzymes as therapeutic adjuncts. At this point, few investigators have applied present day knowledge of tumor enzymology to problems that the physician and his patient face in human cancer. Applied tumor enzymology is undeveloped.

* Elase—Parke, Davis and Company (registered trademark for fibrinolysin-deoxyribonuclease combination).

† Chymotrypsin—Armour Pharmaceutical Company (registered trademark for chymotrypsin-trypsin combination).

Richterich presented the first full report on the clinical application of enzymes in 1958. Preparatory to utilizing enzymes in cancer therapy effectively, intermediate metabolism must be further explored, especially tryptophan metabolism, and an interrelationship with carcinogens and enzyme systems programmed.

Although not directly dealing with enzymes in every case, the publications of certain researchers may serve as general reference ; such as the intermediate metabolism technics of Dalgliesh (1952) Synge and Tiselius (1949) and Robinson *et al.* (1957) ; the cyto-immunological systems of Björklund *et al.* (1957, 1958) ; the clinical laboratory methodology of Seligson (1961), Natelson (1961) and Annino (1960) ; the electron microscope applications of Fisher and Fisher (1961) ; the physical chemistry of Endahl and Kochakian (1957) and Klinkhamer and Eichel (1962) ; the antigen studies of Abramoff *et al.* (1959), Korngold (1960), Weiler (1959), Witebsky *et al.* (1956), and Zilber (1958) ; the specialized metabolic conditions of Beutler (1959), Wheeler and Alexander (1961), Boyland and Watson (1956), Bro-Rasmussen (1958), Lewis *et al.* (1959), and Strominger (1960) ; the comparative chemotherapeutic reports of Von Euler *et al.* (1937), Mirand *et al.* (1961), Hayashi and Fishman (1961) and Cobb *et al.* (1961) ; the reports on carcinogenesis of Elson (1958), Fukui *et al.* (1961), Reid (1962), Kizer (1962), Kizer and Chan (1961), Knox (1960) and Warburg *et al.* (1924). Also, the list could continue and single out references to enzymatic methods for non-enzyme substances, as Gjørup *et al.* (1955), Jørgensen and Chen (1956), Prætorius and Poulsen (1953) and Rehell *et al.* (1952) ; references to biochemical procedures as noteworthy examples, like Hayaishi (1962), Heilbronn (1953), Huggins and Smith (1947), Kurnick (1962), Lowry (1957), Smith and Yanofsky (1962), Wieme (1959*b*), Winer and Schwert (1958) and Wolf *et al.* (1957) ; references to certain histochemical determinations as Winslow and Taylor (1960), Gomori (1957), Winslow and Enzinger (1960), Waterman (1940) and Seligman *et al.* (1949) ; references to pharmacology and associated subjects as Johnson *et al.* (1954), Heath *et al.* (1958), Storm and Nielsen (1958), Timonen and Schroderus (1953) and Werner and Mutt (1954) ; references to immunology as Nairn *et al.* (1960) ; references to carbohydrate synthesis as Gaines (1960) ; references to tumor-host relationship factors as Grace and Lehoczky (1959) ; and more generalized references, as the effect of physical stress on enzymes reported by Halonen and Konttinen (1962), patterns and forms of enzymes reported by Markert and Möller (1959), employment of computer analysis as a new diagnostic tool reported by Mason *et al.* (1961), discovery of a new " enzyme " reported by Murray (1961), speculation on a probable peptidase reported by Pearse and Pepler (1957), discussion of pancreas function tests reported by Popper and Necheles (1959), presentation of lipid haptens reported by Rapport (1961), survey of serum enzymes in disease reported by Rose *et al.* (1961), further information on fibrinolysis reported by Tagnon *et al.* (1953) and further information on seminal plasma by Rhodes and Williams-Ashman (1960).

Anabolic or catabolic enzymes or coenzymes participate in every tumor according to contemporary cancer concepts whether normal, above normal or below normal activity levels. The low level may be even the absence of the enzyme. Apparently, processes to restore when subnormal and destroy or counteract when elevated, and possibly control when normal, by exogenous means, are the outline for productive research in cancer enzymology and therapeutic influence of enzyme activity.

### DISCUSSION OF TRENDS

In the annals of cancer enzymology, there is a continued expansion of reliable analytic procedures designed for both routine and research requirements which is certain to accumulate into an adult science ; equivalent, rather than subordinate to medical biochemistry. When more investigation furnishes data on all phases of each enzyme, much should be contributed to understanding cancer. The metabolic pathways and their affiliated enzymes are yet to be understood, but progress, even though slow, is being made.

Also, there is much effort being made to understand enzyme reactions, specificity and behaviour, coupled with more specific definition of same.

In making a closer examination of emphasized trends, it appears that most, pronounced is the effort to demonstrate isoenzymes and link them with the specific tissues from which they derive. Qualitative and quantitative data have then the added feature of organ derivation ; and cellular differentiation is of tremendous value to any clinician, positively assuring the emergence of enzymology with new existence, new potential and new importance.

There are scattered trends towards understanding the role of enzymes in homeostasis, toward the more extensive usage of enzymes as therapeutic agents, toward human application of certain successful animal studies, toward nutritional effects on enzymes, toward isolated kinetic studies in pure chemistry unrelated to disease, toward evaluation of tumor growth and/or efficacy of treatment, and toward unifying the science, when the contemporary status of enzymes is considered. It is established at this stage that differences exist among tissues and organs (Abramoff *et al.*, 1959 ; Björklund and Björklund, 1957 ; Björklund *et al.*, 1958 ; Grace and Lehoczky, 1959 ; Korngold, 1960 ; Witebsky *et al.*, 1956 ; Zilber, 1959). Most current enzymologists go further and state that all intracellular enzymes are built into a specific and precise structure and that their function depends to a large extent on organization (Green and Järnefelt, 1959). Deoxyribonucleic acid synthesis continuing incessantly, although at vastly different rates, provides the only known clue to understanding the real difference between cancer cells and normal cells. Enzymorphology (Fishman *et al.*, 1961) must be drawn upon to fortify the chemist. More dissension occurs if the report of Angeletti *et al.* (1960), is postulated which claims that tumor proteins resemble one another closely, and, regardless of the original tissue from which they arise, tumors would show essentially similar enzyme patterns. However, almost all agree, the cancer cell, like the normal cell, is exceedingly complex (Weinhouse, 1960).

Nikkilä *et al.* (1960) reported from their studies with erythrocyte enzymes that enzyme defects may be concerned in the shortened survival of red blood cells in patients with nephrogenic and neoplasmatic anemia. They studied nine enzymes on 55 normal subjects and patients with various anemia conditions.

Frei *et al.* (1961) reported from their studies with leukemic cells and normal cells that the monocytes, derived directly from the reticulo-endothelial system, have glycolytic and proteolytic activity, reducing power and esterase activity markedly greater than that of the neutrophilic polynuclear cells. They stated that the lymphocytes showed low active enzymatic functions in comparison to the neutrophil. The eosinophil was reported in pathologic cases resembling the neutrophil in glycolytic activity, but possessing very low proteolytic capacity and oxygen consumption. Frei and his associates reported the enzymatic behaviour of the leukemic cell showed diminished proteolytic power in the myeloblast, but raised glycolysis and occasionally increased esterase activity ; in the lymphoblast, low glycolysis.

Introzzi *et al.* (1961), claimed that metabolism of leucocytes from patients with all forms of leukemia were deficient as shown by a reduction in almost all of the glycolytic enzymes tested.

Fisher and Fisher (1961) disclosed from their study of eight enzymes in carcinomatous tissue by several methods, evidence for proving altered metabolic pathways in neoplastic tissue.

Bodansky (1959) reported that some enzymes in the serum, such as alkaline phosphatase or transaminases, are mixtures of enzymes derived from several tissues.

Cryogenics and cryohomogenation offer a new tool for the understanding of multi-enzyme systems and metabolic pathways and provide a new foundation for the preparation and study of isolated enzyme systems (Klinkhamer and Eichel, 1962).

An uninvestigated possibility is one proposed by Fylling (1961) that fetal growth and metabolic processes increase enzyme activity in the same way as rapidly growing neoplastic tissues, this offered as a possible explanation for enzyme-rich placenta rapidly degenerating at term.

Fishman (1960*a*) expressed his current goal of investigation as being the ability to identify in the circulation the tissue origin of an enzyme by its " marks of identification " or response to specific inhibitors, and said a degree of success has been achieved with acid phosphatase by measuring tartrate-sensitive and insensitive moieties and that this has improved clinical accuracy in cancer of the prostate of the serum ACP determination.

There is some trend toward applying more profoundly chemotherapeutic efforts on the basis of enzymatic lesions revealed (Bergel, 1961 ; Weber and Cantero, 1960) and the attempts are what may be called enzyme pharmacology, forming part of a promising line of cancer research (Weber, 1961).

Does the report of Halonen and Konttinen (1962) on effects of strenuous, but not exhausting, exercise in normal human subjects causing pathological velues in serum LDH and aldolase activity for temporary periods really have clinical significance? Certainly clarification of this mechanism is important.

Mason *et al.* (1961), in a study of urinary enzyme excretion, have pointed out that experimental medicine is extremely complicated because of the large number of interrelated variables which must be considered. Modern computers have the capacity to handle complicated analysis, store data and results of analyses, plot curves, and otherwise report results, modify data and reanalyze, until an equation is reached that logically accounts for the variation observed. Analysis of variance, correlation, and multiple regression analysis techniques are ideally suited for such work, and computer programs are available. Because of the nature of the problems involved in experimental medicine and the capabilities of modern digital computers, it would appear that the growth, probably exponential, of computer use by physicians in research is assured.

There are many technics in common use currently for characterization of enzymes and enzyme reactions ; including automatic chromatographic analysis of amino acid mixtures, density gradient ultracentrifugation of macromolecules,

" finger-printing " of protein digests, optical rotatory dispersion of proteins, gas phase chromatography, gel electrophoresis, immuno-electrophoresis, nuclear magnetic resonance, electron spin resonance, and stop-flow technics for rapid reaction rates. For studies on enzyme content of different cell types, new technics of mammalian cell culture and of histochemistry are now available. In recent years there has occurred a culmination of efforts to elucidate pathways of enzymatic synthesis of all major types of biological macromolecules, including the proteins, nuclei acids, steroids, phospholipids, polysaccharides and porphyrins (Colowick and Kaplan, 1962).

No statement has been made so far in regard to laboratory studies as proof for a given factor to produce cancer in man (Wynder, 1961) but this is an ultimate goal for attainment and one in which the involvement of enzymology should be understood. It is desirable that methods which stand closer to medicine than chemistry in their structure be increasingly employed in clinical investigations (Wuhrmann and Wunderly, 1960).

SUMMARY

This review has attempted to cover the wide range of data accumulated in cancer enzymology with special emphasis on current status and current direction. Employing the newly approved terminology, all enzymes pertinent to neoplastic disease in their respective biochemical classification are discussed. Influencing factors on enzyme activity, carcinogenesis, carcino-chemotherapy and research trends are discussed. Enzymes have been presented with their role in cancer diagnosis, in cancer therapy, in cancer etiology, in the history of cancer and with the intention of pointing out the value of enzymology in tumorigenesis. Prime consideration has been given those correlations which are illustrative of the larger problems to face in cancer definition.

The International Union of Biochemistry has the incorporation of its enzyme commission recommendations in this review. For clarity, whenever possible the popular name, new trivial name and an assigned abbreviation are employed.

Trends are cited and unexplained enzyme characteristics are noted. Methodology is surveyed and new analytical technics are presented. An optimistic view is proffered concerning the development of practical methods for the diagnosis of cancer and the understanding of mechanisms involved. The trend at present seems to be delineation of the growth or regression of the tumor, more than the qualitative or identification testing for cancer, in enzyme determinations.

This review has been as comprehensive, yet concise, as possible. Length and accentuation of certain enzyme topics herein attest to assessed importance in cancer enzymology.

I am grateful to a number of colleagues, particularly Professor Mogens Faber for valuable suggestions and discussions related to this work.

### REFERENCES

Abramoff, P., Chinchinian, H. and Saunders, J. W.—(1959) *J. nat. Cancer Inst.*, **22**, 919.

Abul-Fadl, M. A. M. and King, E. J.—(1948) *J. clin. Path.*, **1**, 80.

Adams, D. H. and Berry, M. E.—(1956) *Biochem. J.*, **64**, 492.

ALLARD, C., DE LAMIRANDE, G. AND CANTERO, A.—(1957) *Cancer Res.*, **17**, 862.
ANGELETTI, P. U., MOORE, B. W. AND SUNTZEFF, V.—(1960) *Ibid.*, **20**, 1592.
ANLYAN, A., GAMBLE, J. AND HOSTER, H.—(1950) *Cancer*, **3**, 116.
ANNINO, J. S.—(1960) 'Clinical Chemistry' (2nd edition). London (Churchill), pp. 232–58.
ASPEN, A. J. AND MEISTER, A.—(1958) in 'Methods of Biochemical Analysis', edited by Glick, D., Vol. VI. New York (Interscience Publishers), pp. 131–61.
ASTRUP, T.—(1956) *Blood*, **11**, 781.
*Idem* AND ALBRECHTSEN, O. K.—(1957) *Scand. J. clin. Lab. Invest.*, **9**, 233.
*Idem* AND STERNDORFF, I.—(1955) *Ibid.*, **7**, 239.
AUGUSTINSSON, K. B.—(1955) *Ibid.*, **7**, 284.
BAKER, R. AND GOVAN, D.—(1953) *Cancer Res.*, **13**, 141.
BAKER, R. W. R. AND PELLEGRINO, C.—(1954) *Scand. J. clin. Lab. Invest.*, **6**, 94.
BARNES, B. A. AND COPE, O.—(1961) *J. Amer. med. Ass.*, **178**, 566.
BECK, W. S.—(1958) *Ann. N.Y. Acad. Sci.*, **75**, 4.
BENNETT L. L., SKIPPER, H. E., SIMPSON, L., WHEELER, G. P. AND WILCOX, W. S.—(1960) *Cancer Res.*, **20**, 62.
BENSON, W. R.—(1957) *Cancer*, **10**, 1235.
BERENBLUM, I.—(1956) *Cancer Res.*, **16**, 675.
BERGEL, F.—(1956) 'Some Chemical Aspects of Abnormal Growth. Lectures on Scientific Basis of Medicine'. London. (Athlone Press).—(1960) *Acta. Un. int. Cancr.*, **16**, 979.—(1961) 'Chemistry of Enzymes in Cancer'. Springfield, Illinois (Thomas).
BEST, C. H. AND TAYLOR, N. B.—(1961) 'The Physiological Basis of Medical Practice', 7th Edition, Baltimore (Williams and Wilkins Co.), p. 667.
BEUTLER, E.—(1959) *J. clin. Invest.*, **38**, 1605.
BIERMAN, H. R., HILL, B. R., EMORY, E., REINHARDT, L. AND SAMUELS, A.—(1955) *Proc. Amer. Ass. Cancer Res.*, **2**, 5.
*Idem*, HILL, B. R., REINHARDT, L. AND EMORY, E.—(1957) *Cancer Res.*, **17**, 660.
BILLOW, B. W., CABODEVILLE, A. M., STERN, A., PALM, A., ROBINSON, M. AND PALEY, S. S.—(1960) *Sthwest Med.*, XLI, 5.
BJÖRKLUND, B. AND BJÖRKLUND, V.—(1957) *Int. Arch. Allergy, N.Y.*, **10**, 153.
*Idem*, LUNDBLAD, G. AND BJÖRKLUND, V.—(1958) *Ibid.*, **12**, 241.
BLANCHAER, M. C., GREEN, P. T. MACLEAN, J. P. AND HOLLENBERG, M. J.—(1958) *Blood*, **13**, 245.
BOCK, J.—(1954) *Scand. J. clin. Lab. Invest.*, **6**, 237.
BODANSKY, O.—(1954*a*) in 'Symposia on Research Advances Applied to Medical Practice, No. 1.' edited by Homburger, F. and Fishman, W. H. 'The Laboratory Diagnosis of Cancer of the Prostate.' Boston (Brown and Conolly). —(1954*b*) *Cancer*, **7**, 1191.—(1954*c*) *Ibid.*, **7**, 1200.—(1955) *Ibid.*, **8**, 1087.—(1956) *Med. Clin. N. Amer.* **40**, 611.—(1957*a*) *Cancer*, **10**, 859.—(1957*b*) *Ibid.*, **10**, 865. —(1958) *Ann. N.Y. Acad. Sci.*, **75**, 380.—(1959) *Amer. J. Med.*, **27**, 861.—(1961) in 'Advances in Cancer Research', edited by Haddow, A. and Weinhouse, S. New York (Academic Press), Vol. 6, pp. 1–80.
BOLIO-CICERO, A., AGUIRRE, J. AND PÉREZ-TAMAYO, R.—(1961) *Amer. J. clin. Path.*, **36**, 417.
BONHAM, D. G. AND GIBBS, D. F.—(1962) *Brit. med. J.*, ii, 823.
BONNER, C. D., HOMBURGER, F. AND FISHMAN, W. H.—(1954) *Surg. Gynec. Obstet.*, **99**, 179.
BORGSTRÖM, B.—(1957) *Scand. J. clin. Lab. Invest.*, **9**, 226.
BOVER, G. F. AND CANDELA, R. B.—(1961) *Rev. clin. esp.*, **83**, 332.
BOYER, S. H.—(1961) *Science*, **134**, 1002.
BOYLAND, E.—(1958) *Brit. med. Bull.*, **14**, 153.
*Idem*, GASSON, J. E. AND WILLIAMS, D. C.—(1957) *Brit. J. Cancer*, **11**, 120.

Boyland, E. and Watson, G.—(1956) *Nature, Lond.*, **177**, 837.
Brandborg, L. L., Taniguchi, L. and Rubin, C. E.—(1961) *Cancer*, **14**, 1074.
Braunstein, H.—(1962) *Ibid.*, **15**, 184.
*Idem*, Freiman, D. G. and Gall, E. A.—(1958) *Ibid.*, **11**, 829.
*Idem*, Freiman, D. G., Thomas Jr., W. and Gall, E. A.—(1962*a*) *Ibid.*, **15**, 130.—(1962*b*) *Ibid.*, **15**, 139.
Bridgewater, A. B. and Necheles, H.—(1957) *Proc. Soc. exp. Biol., N.Y.*, **95**, 84.
Brody, S.—(1958) *Nature, Lond.*, **182**, 1386.
*Idem* and Balis, M. E.—(1958) *Ibid.*, **182**, 940.
Bro-Rasmussen, F.—(1958) *Nutr. Abstr. Rev.*, **28**, 369.
Brüggemann, J., Schlossmann, K., Merkenschlager, M. and Waldschmidt, M.—(1962) *Biochem. Z.*, **335**, 392.
*Idem* and Waldschmidt, M.—(1962) *Ibid.*, **335**, 408.
Bruns, F. F. and Jacob, W.—(1954) *Klin. Wschr.*, **32**, 1041.
Burstone, M. S.—(1958) *J. nat. Cancer Inst.*, **21**, 523.
Candela, R. B. and Bover, G. F.—(1961) *Rev. clin. esp.*, **82**, 324.
Cantero, A.—(1955) in ' Canadian Cancer Conf. '. edited by Begg, R. W. **1**, 309. New York (Academic Press).
Chan, S., McCoy, T. A. and Kizer, D. E.—(1960) *Cancer Res.*, **20**, 1303.
Chevillard, L.—(1945) *C. R. Soc. Biol., Paris*, **139**, 249.
Christensen, L. K.—(1957*a*) *Scand. J. clin. Lab. Invest.*, **9**, 377.—(1957*b*) *Ibid.*, **9**, 380.
Cigarroa, L. G.—(1960) *J. int. Coll. Surg.*, **34**, 442.
Cobb, J. P., Walker, D. G. and Wright, J. C.—(1961) *Cancer Res.*, **21**, 583.
Cohen, S. L. and Huseby, R. A.—(1951*a*) *Ibid.*, **11**, 52.—(1951*b*) *Proc. Soc. exp. Biol., N.Y.*, **76**, 304.
Cohn, C. and Kaplan, A.—(1960) in ' A Textbook of Clinical Pathology ' edited by Miller, S. E., 6th edition, Baltimore (Williams and Wilkins Co.) p. 299.
Colowick, S. P. and Kaplan, N. O.—(1955) ' Methods in Enzymology '. Vol. I. New York (Academic Press).—(1962) *Ibid.* Vol. V. New York (Academic Press).
Dalgliesh, C. E.—(1952) *Biochem. J.*, **52**, 3.
Dawson, R. M. C., Elliott, D. C., Elliott, W. H. and Jones, K. M.—(1959) ' Data for Biochemical Research ', London (Oxford University Press) pp. 148–55.
De, P., Chatterjee, R. and Mitra, S.—(1962) *J. Histochem. Cytochem.*, **10**, 6.
Dean, A. L. and Woodard, H. Q.—(1947) *J. Urol.*, **57**, 172.
De Lamirande, G. and Allard, C.—(1959) *Ann. New York Acad. Sci.*, **81**, 570.
*Iidem* and Cantero, A.—(1958) *Cancer Res.*, **18**, 952.
Desnuelle, P.—(1961) ' The Enzymes of Lipid Metabolism ', Oxford (Pergamon Press).
Dioguardi, N., Agostino, A., Lomanto, B., Doniselli, M., Maroni, G. C., Febbri, F. and Schweizer, M.—(1961*b*) *Academia Media Lombarda*, Atti **16**, 166.
*Idem*, Agostino, A., Tittobello, A., Fiorelli, G. and Lomanto, B.—(1961*a*) *Ibid.*, Atti **16**, 159.
Dixon, M. and Webb, E. C.—(1958) ' Enzymes ' London (Longmanns, Green and Co.).
Dixon, T. F. and Purdom, M.—(1954) *J. clin. Path.*, **7**, 341.
Dodgson, K. S. and Spencer, B.—(1957) *Biochem. J.*, **65**, 668.
*Iidem* and Wynn, C. H.—(1956) *Ibid.*, **62**, 500.
Dux, C., Guerin, M. and Lacour, F.—(1948) *C. R. Soc. Biol., Paris*, **142**, 789.
Dybkær, R. and Jensen, G.—(1958) *Scand. J. clin. Lab. Invest.*, **10**, 349.
Dzialoszynski, L. M.—(1957) *Clin. Chim. Acta*, **2**, 542.
Eastham, R. D.—(1960) ' Biochemical Values in Clinical Medicine ', Bristol (John Wright).
Ekman, B., Thune, S. and Truedsson.—(1953) *Scand. J. clin. Lab. Invest.*, **5**, 175.

ELLIOTT, B. A. AND WILKINSON, J. H.—(1961) *Lancet*, i, 698.
ELSON, L. A.—(1958) *Brit. med. Bull.*, **14**, 161.
ENDAHL, G. L. AND KOCHAKIAN, C. D.—(1957) *Proc. Soc. exp. Biol., N.Y.*, **94**, 192.
ENDE, N.—(1961) *Cancer*, **14**, 1109.
FABER, V. AND SCHMITH, K.—(1950*a*) *Scand. J. clin. Lab. Invest.*, **2**, 298.—(1950*b*) *Ibid.*, **2**, 303.
FARBER, S.—(1955) *Trans. Coll. Physicians, Phila.*, **4**, 74.
FEINSTEIN, R. N. AND VETTER, M.—(1961) in ' Argonne National Laboratory, Biological and Medical Division, Summary Report for 1960 '. Chicago (University of Chicago).
FENTON, P. F.—(1960) in ' Medical Physiology and Biophysics ', edited by Ruch, T. C. and Fulton, J. F., 18th edition. Philadelphia (W. B. Saunders Co.) pp. 909–11.
FIESSINGER, N. AND BOYER, F.—(1935) *Rev. méd.-chir. Mal. Foie*, **10**, 137.
FIGGE, F. H. J. AND STRONG, L. C.—(1941) *Cancer Res.*, **1**, 779.
FISCHER, F. AND SIEBERT, B.—(1961) *Klin. Wschr.*, **39**, 202.
FISHER, E. R. AND FISHER, B.—(1961) *Cancer Res.*, **21**, 527.
FISHMAN, W. H.—(1949) *Bull. New Engl. med. Cent.* **11**, 235.—(1960*a*) in ' The Physiopathology of Cancer ', edited by Homburger, F., 2nd edition, New York (Paul B. Hoeber, Inc.) pp. 732–60.—(1960*b*) in ' The Plasma Protein ', edited by Putnam, F. W. New York (Academic Press) Vol. 2, pp. 59–103.
*Idem* AND ANLYAN, A. J.—(1947) *J. biol. Chem.*, **169**, 449.
*Idem*, ANLYAN, A. J. AND GORDON, E.—(1947) *Cancer Res.*, **7**, 808.
*Idem*, BAKER, J. R. AND BORGES, P. R. F.—(1959) *Cancer*, **12**, 240.
*Idem* AND BIGELOW, R. M.—(1950) *J. nat. Cancer Inst.*, **10**, 1115.
*Idem*, BONNER, C. D. AND HOMBURGER, F.—(1956) *New Engl. J. Med.*, **255**, 925.
*Idem*, DART, R. M., BONNER, C. D., LEADBETTER, W. F., LERNER, F. AND HOMBURGER, F.—(1953) *J. clin. Invest.*, **32**, 1034.
*Idem* AND DAVIDSON, H. M.—(1957) in ' Methods of Biochemical Analysis ' edited by Glick, D. New York (Interscience Publishers) Vol. IV, pp. 257–84.
*Idem*, GREEN, S. HOMBURGER, F., KASDON, S. C., NIEBURGS, H. E., MCINNIS, G. AND PUND, E. R.—(1954) *Cancer*, **7**, 729.
*Idem*, LADUE, K. T. AND BORGES, P. R. F.—(1961) *J. Histochem. Cytochem.*, **9**, 424.
*Idem* AND LERNER, F.—(1953) *J. biol. Chem.*, **200**, 89.
*Idem* AND MITCHELL, G. W —(1959) *Ann. N.Y. Acad. Sci.*, **83**, 105.
*Idem*, SPRINGER, B. AND BRUNETTI, R.—(1948) *J. biol. Chem.*, **173**, 449.
FITZPATRICK, T. B.—(1952) *Arch. Derm. Syph., Chicago*, **65**, 379.
FLETCHER, A. P.—(1954) *Biochem. J.*, **56**, 677.
FORSELL, O. M. AND PALVA, I. P.—(1961) *Scand. J. clin. Lab. Invest.*, **13**, 131.
FRAENKEL-CONRAT, H.—(1957) in ' Methods in Enzymology ' edited by Colowick, S. P. and Kaplan, N.O. New York (Academic Press) Vol. IV, pp. 247–69.
FRANSEEN, C. C. AND MCLEAN, R.—(1935) *Amer. J. Cancer*, **24**, 299.
FREEMAN, M. E.—(1961) *Scand. J. clin. Lab. Invest.*, **13**, 346.
FREI, J., BOREL, Cl., HORVATH, G., CULLITY, B. AND VANNOTTI, A.—(1961) *Blood*, **18**, 317.
FRIEDMAN, M. M. AND BECKER, E.—(1955) *Clin. Chem.*, **1**, 110.
FUKUI, K., NAGATA, C., IMMAMURA, A. AND TAGASHIRA, Y.—(1961) *Gann*, **52**, 127.
FYLLING, P.—(1961) *Scand. J. clin. Lab. Invest.*, **13**, 264.
GAINES, JR., L. M.—(1960) *Johns Hopk. Hosp. Bull.*, **106**, 195.
GAL, E. M. AND ROTH, E.—(1957) *Clin. Chim. Acta*, **2**, 316.
GJØRUP, S., POULSEN, H. AND PRÆTORIUS, E.—(1955) *Scand. J. clin. Lab. Invest.*, **7**, 201.
GLICK, D.—(1959) in ' The Cell ', edited by Brachet, J. and Mirsky, A. E. New York (Academic Press) Vol. I, pp. 151–53.
GOLDBERG, J. A. AND RUTENBURG, A. M.—(1958) *Cancer*, **11**, 283.

GOMORI, G.—(1941) *Arch. Path.*, **32**, 189.—(1957) in ' Methods in Enzymology ' edited by Colowick, S. P. and Kaplan, N. O. New York (Academic Press) Vol. IV. pp. 381–91.
GRACE, JR., J. T. AND LEHOCZKY, A.—(1959) *Surgery*, **46**, 238.
GRAY, C. H.—(1959) ' Clinical Chemical Pathology ', (2nd edition) London (Edward Arnold, Ltd) p. 98.
GRAY, S. J., RAMSEY, C. G., REIFENSTEIN, R. W. AND KRAKAUER, L. J.—(1955) *Gastroenterology*, **29**, 641.
GREEN, D. E. AND JÄRNEFELT, J.—(1959) *Perspect. Biol. Med.*, **2**, 163.
GREEN, H. N. AND JENKINSON, C. N.—(1934) *Brit. J. exp. Path.*, **15**, 1.
GREEN, J. B.—(1958) *J. nerv. ment. Dis.*, **127**, 359.
GREENSTEIN, J. P.—(1954) ' Biochemistry of Cancer ', (2nd edition) New York (Academic Press).—(1956) *Cancer Res.*, **16**, 641.
*Idem*, WERNE, J., ESCHENBRENNER, A. B. AND LENTHARDT, F. M.—(1944) *J. nat. Cancer Inst.*, **5**, 55.
GRIBOFF, S. I., HERRMAN, J. B., SMELIN, A. AND MOSS, J.—(1954) *J. clin. Endocrin.*, **14**, 378.
GRÖNVALL, C.—(1961) *Scand. J. clin. Lab. Invest.*, **13**, 29.
GUTMAN, A. B.—(1959) *Amer. J. Med.*, **27**, 875.
*Idem* AND GUTMAN, E. B.—(1938) *J. clin. Invest.*, **17**, 473.
*Iidem* AND ROBINSON, J. N.—(1940) *Amer. J. Cancer*, **38**, 103.
GUTMAN, E. B. AND GUTMAN, A. B.—(1940) *J. biol. Chem.*, **136**, 201.
*Idem*, SPROUL, E. E. AND GUTMAN, A. B.—(1936) *Amer. J. Cancer*, **28**, 485.
HALONEN, P. I. AND KONTTINEN, A.—(1962) *Nature, Lond.*, **193**, 942.
HANSEN, H. AND JACOBSSON, L.—(1952) *Scand. J. clin. Lab. Invest.*, **4**, 183.
HARPER, H. A.—(1958) *Annu. Rev. Med.*, **9**, 461.
HAYASHI, M. AND FISHMAN, W. H.—(1961) *Acta endocr., Copenhagen*, **38**, 107.
HAYAISHI, O.—(1962) in ' Methods in Enzymology ', edited by Colowick, S. P. and Kaplan, N. O. New York (Academic Press) Vol. V. pp. 807–809.
HEATH, R. G., LEACH, B. E., BYERS, L. W., MARTENS, S. AND FEIGLEY, C. A.—(1958) *Amer. J. Psychiat.* **114**, 683.
HEILBRONN, A.—(1953) *Scand. J. clin. Lab. Invest.*, **5**, 308.
HENLEY, K. S. AND POLLARD, H. M.—(1955) *J. Lab. clin. Med.*, **46**, 785.
HERBERT, F. K.—(1946) *Quart. J. Med.* (New Series), **15**, 221.
HILL, B. R.—(1958) *Ann. N.Y. Acad. Sci.*, **75**, 304.—(1961) *Cancer Res.*, **21**, 271.
*Idem* AND JORDAN, R. T.—(1957) *Ibid.*, **17**, 144.
*Idem*, KUFF, E. L. AND HOGEBOOM, G. H.—(1956) *Proc. Soc. exp. Biol. N.Y.*, **92**, 430.
*Idem* AND LEVI, C.—(1954) *Cancer Res.*, **14**, 513.
HOCK, E. AND TESSIER, R. N.—(1949) *J. Urol.*, **62**, 488.
HODSON, A. W., LATNER, A. L. AND RAINE, L.—(1962) *Clin. Chim. Acta.*, **7**, 255.
*Iidem* AND SKILLEN, A. W.—(1961) *J. Physiol.*, **159**, 54 P.
HOFFMAN, G. T., ROTTINO, A. AND STERN, K. G.—(1951) *Blood*, **6**, 1051.
HOLMBERG, G. C. G. A? D LAURELL, C. B.—(1951) *Scand. J. clin. Lab. Invest.*, **3**, 103.
HOMBURGER, F.—(1960) ' The Physiopathology of Cancer ', 2nd edition, New York (P. B. Hoeber, Inc.) pp. 896–98.
*Idem* AND FISHMAN, W. H.—(1956) ' The Laboratory Diagnosis of Cancer of the Cervix.' Symposia on Research Advances Applied to the Practice of Medicine, No. 2, Basel and New York (S. Karger).
HOUCK, J. C.—(1958) *J. appl. Physiol.*, **13**, 273.
HSIEH, K. M. AND BLUMENTHAL, H. T.—(1956) *Proc. Soc. exp. Biol. N.Y.* **91**, 626.
*Idem*, SUNTZEFF, V. AND COWDRY, E. V.—(1955) *Ibid.*, **98**, 627.—(1956) *Cancer Res.*, 16, 237.
HUDSON, P. B., TSUBOI, K. K. AND MITTELMAN, A.—(1955) *Amer. J. Med.*, **19**, 895.
HUGGINS, C. AND HODGES, C. V.—(1941) *Cancer Res.*, **1**, 293.

HUGGINS, C. AND SMITH, D. R.—(1947) *J. biol. Chem.*, **170**, 391.
HUMOLLER, F. L., MAJKA, F. A., BARAK, A. J., STEVENS, J. D. AND HOLTHAUS, J. M.—(1958) *Clin. Chem.*, **4**, 1.
INTROZZI, P., NOTARIO, A. AND NESPOLI, M.—(1961) *Haematologica*, **46**, 401.
ISRAELS, L. G. AND DELORRY, G. E.—(1956) *Brit. J. Cancer*, **10**, 318.
IWATSURU, R. AND MINAMI, Y.—(1934) *Biochem. Z.*, **268**, 394.
*Idem* AND NANJO, K.—(1939) *Ibid.*, **300**, 422.
JACOBSSON, L. AND HANSEN, H.—(1952) *Scand. J. clin. Lab. Invest.*, **4**, 134.
JAGT, T. AND LARSEN, O. A.—(1960) *Ibid.*, **12**, 200.
JØRGENSEN, K. AND SVENDSEN, A.—(1961) *Ibid.*, **13**, 122.
JØRGENSEN, M. B.—(1954) *Ibid.*, **6**, 303.
JØRGENSEN, S. AND CHEN JR., P. S.—(1956) *Ibid.*, **8**, 145.
JOHNSON, A. J., GOGER, P. R. AND TILLETT, W. S.—(1954) *J. clin. Invest.*, **33**, 1670.
JOSEFSSON, L. AND LAGERSTEDT, S.—(1962) in ' Methods of Biochemical Analysis ', edited by Glick, D., New York (Interscience Publishers) Vol. IX, pp. 39–74.
KÄRCHER, H. K.—(1962) *Ärztl. Forsch.*, **16**, 38.
KARMEN, A., WROBLEWSKI, F. AND LADUE, J. S.—(1955) *J. clin. Invest.*, **34**, 126.
KASDON, S. C., FISHMAN, W. H. AND HOMBURGER, F.—(1950) *J. Amer. med. Ass.*, **144**, 892.
*Idem*, HOMBURGER, F., YORSHIS, E. AND FISHMAN, W. H.—(1953) *Surg. Gynec. Obstet.*, **97**, 579.
KAUFMAN, N. AND HILL, R. W.—(1960) *Cancer Res.*, **20**, 335.
KAY, H. D.—(1930) *J. biol. Chem.*, **89**, 235.
KEIDING, N. R.—(1959) *Scand. J. clin. Lab. Invest.*, **11**, 106.
KERPPOLA, W.—(1951) *Blood*, **6**, 454.
KIDSON, C.—(1962) *Ibid.*, **19**, 82.
KING, E. J.—(1957) *Clin. Chem.*, **3**, 507.—(1959) *Amer. J. Med.*, **27**, 849.
*Idem* AND CAMPBELL, D. M.—(1961) *Clin. Chim. Acta*, **6**, 301.
KIRKEBY, K. AND PRYDZ, H.—(1959) *Scand. J. clin. Lab. Invest.*, **11**, 185.
KIT, S.—(1960) *Cancer Res.*, **20**, 1121.
KIZER, D. E.—(1962) *Ibid.*, **22**, 196.
*Idem* AND CHAN, S. K.—(1961) *Ibid.*, **21**, 489.
KLINKHAMER, J. M. AND EICHEL, B.—(1962) *Nature, Lond.*, **193**, 944.
KNOX, W. E.—(1960) *Acta. Un. int. Cancr.*, **16**, 1018.
KORNGOLD, L.—(1960) National Cancer Institute Monograph No. 2. Symposium on ' Normal and Abnormal Differentiation and Development ', Bethesda, Maryland, pp. 57–71.
KOWLESSAR, O. D., ALTMAN, K. I. AND HEMPELMANN, L. H.—(1953) *Nature, Lond.*, **172**, 867.
KURNICK, N. B.—(1962) in ' Methods of Biochemical Analysis ' edited by Glick, D. New York (Interscience Publishers) Vol. IX, pp. 1–38.
KUTSCHER, W. AND WOLBERGS, H.—(1935) *Z. physiol. Chem.*, **236**, 237.
LADEHOFF, Å. AND RASMUSSEN, J.—(1961) *Scand. J. clin. Lab. Invest.*, **13**, 231.
LATNER, A. L AND SKILLEN, A. W.—(1961) *Lancet*, ii, 1286.
LAURSEN, T.—(1959) *Scand. J. clin. Lab. Invest.*, **11**, **134**.—(1962) *Ibid.*, **14**, 152.
*Idem* AND ESPERSEN, G.—(1959) *Ibid.*, **11**, 61.
*Idem* AND FROM-HANSEN, P.—(1958) *Ibid.*, **10**, 53.
LEMON, H. M., REYNOLDS, M. D. AND KELLEY, D.—(1958) in ' Proc. International Symposium Mammary Cancer Division of Cancer Research ', University of Perugia, Italy, pp. 99–109.
*Idem* AND WISSEMAN, C. L.—(1949) *Science*, **109**, 233.
LEWIS, K. F., MAJANE, E. H. AND WEINHOUSE, S.—(1959) *Cancer Res.*, **19**, 97.
LEWIS, U. J., WILLIAMS, D. E. AND BRINK, N. G.—(1956) *J. biol. Chem.*, **222**, 705.
LIBENSON, L. AND JENA M.—(1957) *Cancer*, **10**, 1004.

Løken, F.—(1956) *Scand. J. clin. Lab. Invest.*, **8**, 175.
Long, C., King, E. J. and Sperry, W. M.—(1961) ' Biochemists Handbook '. London (E. and F. Spon, Ltd.).
Lowry, O. H.—(1957) in ' Methods in Enzymology ' edited by Colowick, S. P. and Kaplan, N. O. New York (Academic Press) Vol. IV, pp. 366–81.
Lundh, G.—(1957) *Scand. J. clin. Lab. Invest.*, **9**, 229.
Manso, C. and Wroblewski, F.—(1958) *J. clin. Invest.*, **37**, 214.
Margulis, R. R., Ladd, J. E., Fahey, M. F. and Walser, H. C.—(1961) *Amer. J. Obstet. Gynec.*, **81**, 840.
Markert, C. L. and Möller, F.—(1959) *Proc. nat. Acad. Sci., Wash.*, **45**, 753.
Mason, E. E., Chernigoy, F. and Cusminsky, B.—(1961) *J. Amer. med. Ass.*, **178**, 1088.
Mason, R.—(1958) *Brit. J. Cancer*, **12**, 469.
Mather, G., Richmond, S. G. and Sprunt, D. H.—(1956) *J. Urol.*, **75**, 143.
Meijer, A. E. F. H.—(1962) *Biochem. Pharmacol.*, **2**, 125.
Meister, A.—(1950) *J. nat. Cancer. Inst.*, **10**, 1263.
Mendelsohn, M. L. and Bodansky, O.—(1952) *Cancer*, **5**, 1.
Michaelis, L. and Menten, M. L.—(1913) *Biochem. Z.*, **49**, 333.
Mirand, E. A., Back, N., Prentice, T. C., Ambrus, J. L. and Grace Jr., J. T.—(1961) *Proc. Soc. exp. Biol., N.Y.*, **108**, 360.
Morton, R. K.—(1958) *Nature, Lond.*, **181**, 540.
Moss, D. W.—(1962) *Ibid.*, **193**, 981.
Murray, M.—(1961) *Amer. J. clin. Path.*, **36**, 500.
Myers, W. P. L. and Bodansky, O.—(1957) *Amer. J. Med.*, **23**, 804.
Nairn, R. C., Richmond, H. G., McEntegart, M. G. and Fothergill, J. E.—(1960) *Brit. med. J.*, ii, 1335.
Nardi, G. L. and Lees, C. V.—(1958) *New Engl. J. Med.*, **258**, 797.
Natelson, S.—(1961) ' Microtechniques of Clinical Chemistry ', 2nd edition. Springfield, Illinois (Chas. C. Thomas) pp. 93–334.
Neufach, S. A. and Melnikova, M. P.—(1958) *Biochimica*, **23**, 440.
Nigam, V. N., MacDonald, H. L. and Cantero, A.—(1962) *Cancer Res.*, **22**, 131.
Nikkilä, E. A., Pitkänen, E., Vuopio, P. and Forsell, O.—(1960) *Ann. Med. intern. Fenn.*, **49**, 187.
Nisselbaum, J. S. and Bodansky, O.—(1961*a*) *J. biol. Chem.*, **236**, 323.—(1961*b*) *Ibid.*. **236**, 401.
Norberg, B.—(1961) *Clin. Chim. Acta*, **6**, 264.
Nylander, G.—(1955) *Scand. J. clin. Lab. Invest.*, **7**, 254.
Odell, L. D., Burt, J. and Bethea, R.—(1949) *Cancer Res.*, **9**, 362.
Paul, J.—(1962) *Ibid.*, **22**, 431.
Pearse, A. G. E.—(1960) ' Histochemistry ' 2nd edition London (J. and A. Churchill).
*Idem* and Pepler, W. J.—(1957) *Nature, Lond.*, **179**, 589.
Plagemann, P. G. W., Gregory, K. F. and Wroblewski, F.—(1960) *J. biol. Chem.*, **235**, 2288.
Plummer, D. T. and Wilkinson, J. H.—(1962) *Biochem. J.*, **81**, 10.
Popper, H. L. and Necheles, H.—(1959) *Med. Clin. N. Amer.*, **43**, 401.
Posnanskaya, A. A.—(1958) *Biochimica*, **23**, 230.
Potter, V. R.—(1956) *Cancer Res.*, **16**, 658.—(1957) *Univ. Mich. med. Bull.*, **23**, 401.—(1958) *Fed. Proc.*, **17**, 691.
Powell, M. E. A. and Smith, M. J. H.—(1954) *J. clin. Path.*, **7**, 245.
Pravdeena, K. I.—(1957) *Vop. Onkol.*, **3**, 85.
Prætorius, E. and Poulsen, H.—(1953) *Scand. J. clin. Lab. Invest.*, **5**, 273.
Rapp, R. D. and Bell, E. R.—(1961) *Amer. J. clin. Path.*, **35**, 116.
Rapport, M. M.—(1961) *J. Lipid Res.*, **2**, 25.
Rauramo, L.—(1959*a*) *Scand. J. clin. Lab. Invest.*, **11**, 285.—(1959*b*) *Ibid.*, **11**, 290.

Rehell, B., Forsander, O. and Räihä, C. E.—(1952) *Ibid.*, **4**, 211.
Reichard, H.—(1957) *Ibid.*, **9**, 311.
Reichard, P., Sköld, O., Klein, G., Révész, L. and Magnusson, P. H.—(1962) *Cancer Res.*, **22**, 235.
Reid, E.—(1962) *Cancer Res.*, **22**, 398.
Report of the Commission on Enzymes of the International Union of Biochemistry—(1961) I.U.B. Symposium Series Vol. 20. Oxford (Pergamon Press).
Reynolds, M. D., Lemon, H. M. and Byrnes, W. W.—(1956) *Cancer Res.*, **16**, 943.
Rhodes, J. B. and Williams-Ashman, H. G.—(1960) *Med. Exp.* **3**, 123.
Richterich, R.—(1958) ' Enzymopathologie : Enzyme in Klinik und Forschung ', Berlin (Springer-Verlag).
Riley, V. and Wroblewski, F.—(1960) *Science*, **132**, 151.
Robert, J. and Van Rymenant, M.—(1961) *Chimie Pure et Appliquée*, **3**, 475.
*Idem*, Van Rymenant, M. and Lagae, F.—(1961) *Cancer*, **14**, 1166.
Roberts, E.—(1948) *J. biol. Chem.*, **176**, 213.
Robins, E.—(1957) *Exp. Cell Res.*, Suppl. 4, 241.
Robinson, W. P., Nagle, R., Bachhawat, B. K., Kupiecki, F. P. and Coon, M. J.—(1957) *J. biol. Chem.*, **224**, 1.
Roche, J.—(1931) *Biochem. J.*, **25**, 1724.
Rose, A., West, M. and Zimmerman, H. J.—(1961) *Cancer*, **14**, 726.
Ruch, T. C. and Fulton, J. F.—(1960) ' Medical Physiology and Biophysics ', 18th edition. Philadelphia (W. B. Saunders Co.), pp. 1010–14.
Sabine, J. C.—(1951) *Blood*, **6**, 151.
Saffran, M. and Scarano, E.—(1953) *Nature Lond.*, **172**, 949.
Sayre, F. W. and Hill, B. R.—(1957) *Proc. Soc. exp. Biol., N.Y.*, **96**, 695.
Scarano, E.—(1953) *Nature, Lond.*, **172**, 951.
Schenker, S.—(1959) *Amer. J. dig. Dis.* New Series 4, 412.
Schlamowitz, M. and Bodansky, O.—(1959) *J. biol. Chem.*, **234**, 1433.
Schmith, K. and Faber, V.—(1950) *Scand. J. clin. Lab. Invest.*, **2**, 292.
Schwartz, M. K. and Bodansky, O.—(1959) *Proc. Soc. exp. Biol. N.Y.*, **101**, 560.
Seligman, A. M., Nachlas, M. M., Manheimer, L. H., Friedman, O. M. and Wolf, G.—(1949) *Ann. Surg.*, **130**, 330.
Seligson, D.—(1961) ' Standard Methods of Clinical Chemistry ' New York (Academic Press) Vol. 3, pp. 14–22.
Sibley, J. H. and Lehninger, A. L.—(1949) *J. nat. Cancer Inst.*, **9**, 303.
Singer, T. P. and Kearney, E. B.—(1957) in ' Methods of Biochemical Analysis ', edited by Glick, D., New York (Interscience Publishers) Vol. IV., pp. 307–33.
Smith, O. H. and Yanofsky, C.—(1962) in ' Methods in Enzymology ', edited by Colowick, S. P. and Kaplan, N. O. New York (Academic Press) Vol. V., pp. 794-806.
Sørensen, S. P. L.—(1909) *Biochem. Z.*, **21**, 131, 201.
Starkweather, W. H., Schoh, H. K. and Holt, F. J.—(1961) *Clin. Res.*, **9**, 24.
Stave, V. U. and Oehme, J.—(1961/62) *Enzym. Biol. Clin., Basel*, **1**, 75.
Stekol, J. A.—(1958) *Annu. Rev. Biochem.*, **27**, 679.—(1959) *Ibid.*, **28**, 605.
Sterkel, R. L., Spencer, J. A., Wolfson, S. K. and Williams-Ashman, H. G.—(1958) *J. Lab. clin. Med.*, **52**, 176.
Stern, J. R., Campillo, A. and Coon, M. J.—(1956*a*) *J. biol. Chem.*, **221**, 1.—(1956*b*) *Ibid.* **221**, 15.
Storm, O. and Nielsen, M. H.—(1958) *Nord. med.*, **60**, 1831.
Stovner, J.—(1955) *Scand. J. clin. Lab. Invest.*, **7**, 197.
Straub, F. B., Stephaneck, O. and Acs, G.—(1957) *Biochimica*, **22**, 118.
Strauss, B. S.—(1960) ' An Outline of Chemical Genetics '. Philadelphia (W. B. Saunders Co.).
Strominger, J. L.—(1960) *Physiol. Rev.*, **40**, 67.

SYNGE, R. L. M. AND TISELIUS, A.—(1949) *Acta chem. scand.*, **3**, 231.

TAGNON, H. J., WHITMORE, JR., W. F., SCHULMAN, P. AND KRAVITZ, S. C.—(1953) *Cancer*, **6**, 63.

TAMMELIN, L. E.—(1953) *Scand. J. clin. Lab. Invest.*, **5**, 267.

TANAKA, K. R., VALENTIN, W. N. AND FREDRICKS, R. E.—(1962) *Brit. J. Haemat.*, **8**, 86.

THIERS, R. E. AND VALLEE, B. L.—(1958) *Ann. N.Y. Acad. Sci.*, **75**, 214.

THOMPSON, R. H. S.—(1962) *Nature, Lond.*, **193**, 1227.

TILLETT, W. S. AND GARNER, R. L.—(1933) *J. exp. Med.*, **58**, 485.

*Idem*, JOHNSON, A. J. AND MCCARTY, W. R.—(1955) *J. clin. Invest.*, **34**, 169.

TIMONEN, S. AND SCHRODERUS, K. A.—(1953) *Scand. J. clin. Lab. Invest.*, **5**, 207.

TORP, H. E.—(1956) *Ibid.*, **8**, 84.

TRUEDSSON, E.—(1951) *Uppsala LäkFören. Förh.*, **56**, 39.

UDENFRIEND, S., WEISSBACH, H. AND BRODIE, B.—(1958) in ' Methods of Biochemical Analysis ', edited by Glick, D. New York (Interscience Publishers) Vol. VI, pp. 95–130.

VALENTIN, W. N.—(1956) *Progr. Haemat.*, **1**, 293.

VAN RYMENANT, M. AND ROBERT, J.—(1961) *Chimie Pure et Appliquée* **3**, 473.

*Idem* AND *Tagnon*, H. J.—(1959*a*) *New Engl. J. Med.*, **261**, 1325.—(1959*b*) *Ibid.*, **261**, 1373.

VESELL, E. S. AND BEARN, A. G.—(1958) *Ann. N.Y. Acad. Sci.*, **75**, 286.—(1961) *J. clin. Invest.*, **40**, 586.

VETTER, K.—(1961) *Folia Haemat. Frankfurt*, **6**, 80.

*Idem* AND GRIESCHE, H.—(1961) *Acta haemat.*, **26**, 344.

VON EULER, H., MALMBERG, M. AND GUNTHER, G.—(1937) *Z. Krebsforsch.*, **45**, 425.

WACHSTEIN, M.—(1946) *J. Lab. clin. Med.*, **31**, 1.

WAIN, H.—(1958) ' The Story Behind the Word '. Springfield, Illinois (Chas. C. Thomas).

WARBURG, O.—(1956) *Science*, **123**, 309.

*Idem*, POSENER, K. AND NEGELEIN, E.—(1924) *Biochem. Z.* **152**, 309.

WATERMAN, N.—(1940) *Acta brev. neerl. Physiol.*, **10**, 205.

WEBER, G.—(1959) *Rev. canad. Biol.*, **18**, 245.—(1961) in ' Advances in Cancer Research ', edited by Haddow, A. and Weinhouse, S. New York (Academic Press) Vol. 6, pp. 403–494.

*Idem* AND CANTERO, A.—(1960) *Acta Un. int. Cancr.*, **16**, 1002.

WELCH, A. D. —(1961) *Cancer Res.*, **21**, 1475.

WEILER, E.—(1959) in ' Ciba Foundation Symposium on Carcinogenesis ', Boston (Little, Brown and Co.), pp. 165–74.

WEINHOUSE, S.—(1960) *Acta Un. int. Cancr.*, **16**, 32.

WERNER, B. AND MUTT, V.—(1954) *Scand. J. clin. Lab. Invest.*, **6**, 228.

WHEELER, G. P. AND ALEXANDER, J. A.—(1961) *Cancer Res.*, **21**, 407.

WHITE, L. P.—(1958*a*) *J. nat. Cancer. Inst.*, **21**, 671.—(1958*b*) *Ibid.*, **21**, 685.—(1958*c*) *Ann. N.Y. Acad. Sci.*, **75**, 349.

WIEME, R. J.—(1959*a*) *Clin. Chim. Acta*, **4**, 46.—(1959*b*) ' Studies on Agar Gel Electrophoresis Thesis ', Bruxelles (Arscia).

WINER, A. D. AND SCHWERT, G. W.—(1958) *Science*, **128**, 660.

WINKELMAN, R. K.—(1961) *Cancer*, **14**, 1001.

WINSLOW, D. J. AND ENZINGER, F. M.—(1960) *Amer. J. Path.*, **37**, 497.

*Idem* AND TAYLOR, H. B.—(1960) *Cancer*, **13**, 127.

WITEBSKY, E., ROSE, N. R. AND SHULMAN, S.—(1956) *Cancer Res.*, **16**, 831.

WÖRNER, W. AND MARTIN, H.—(1961) *Klin. Wschr.*, **39**, 368.

WOLF, H. P., FORSTER, G. AND LEUTHARDT, F.—(1957) *Gastroenterologia*, **87**, 172.

WOLFSON, S. K., SPENCER, J. A., STERKEL, R. L. AND WILLIAMS-ASHMANN, H. G.—(1958) *Ann. N.Y. Acad. Sci.*, **75**, 260.

WOLFSON, S. K. AND WILLIAMS-ASHMAN, H. G.—(1957) *Proc. Soc. exp. Biol. N.Y.*, **96**, 321.
WOODARD, H. Q.—(1952) *Cancer*, **5**, 236.—(1959) *Amer. J. Med.*, **27**, 902.
WRIGHT, J. C.—(1961) New York State J. Med. **61**, 249.
WROBLEWSKI, F.—(1958*a*) *Ann. N.Y. Acad. Sci.*, **75**, 322.—(1958*b*) in 'Advances in Clinical Chemistry', edited by Sobotka, H. and Stewart, C. R. New York (Academic Press) Vol. I, pp. 313–51.—(1959*a*) *Amer. J. Med.*, **27**, 911.—(1959*b*) *Ann. intern. Med.*, **50**, 62.
*Idem*, DECKER, B. AND WROBLEWSKI, R.—(1957) *Amer. J. clin. Path.*, **28**, 269.—(1958) *New Engl. J. Med.*, **258**, 635.
*Idem* AND LADUE, J. S.—(1955) *Proc. Soc. exp. Biol., N.Y.*, **90**, 210.
*Idem* AND WROBLEWSKI, R.—(1958) *Ann. intern. Med.*, **48**, 813.
WUHRMANN, F. AND WUNDERLY, C.—(1960) 'The Human Blood Proteins'. New York (Grune and Stratton).
WYNDER, E. L.—(1961) *Cancer Res.*, **21**, 858.
XEFTERIS, E., MITUS, W. J., MEDNICOFF, I. B. AND DAMESHEK, W.—(1961) *Blood*, **18**, 202.
YOUNG, I. L.—(1958) *Ann. N.Y. Acad. Sci.*, **75**, 357.
ZILBER, L. A.—(1958) *Advanc. Cancer. Res.*, **5**, 291.—(1959) *Neoplasma*, **6**, 337.
ZUCKER, M. B. AND BORELLI, J.—(1958) *Ann. N.Y. Acad. Sci.*, **75**, 203.—(1959) *J. clin. Invest.*, **38**, 148.